# Mechanistic Insights of Ethylene Polymerization on Phillips Chromium Catalysts

**DOI:** 10.3390/polym16050681

**Published:** 2024-03-02

**Authors:** Ilya Nifant’ev, Pavel Komarov, Guzelia Sadrtdinova, Vadim Safronov, Nikolay Kolosov, Pavel Ivchenko

**Affiliations:** 1 A.V. Topchiev Institute of Petrochemical Synthesis RAS, 29 Leninsky Pr., 119991 Moscow, Russia; komarrikov@yandex.ru (P.K.); gisadrtdinova@edu.hse.ru (G.S.); phpasha1@yandex.ru (P.I.); 2 Department of Chemistry, M.V. Lomonosov Moscow University, 1–3 Leninskie Gory, 119991 Moscow, Russia; 3 Faculty of Chemistry, National Research University Higher School of Economics, Myasnitskaya St. 20, 101100 Moscow, Russia; 4 NIOST LLC, Kuzovlevsky Tr. 2–270, 634067 Tomsk, Russia; safronovva@niost.sibur.ru (V.S.); kolosovna@niost.sibur.ru (N.K.)

**Keywords:** activation, chromium catalysts, coordination polymerization, DFT, mesoporous supports, Phillips catalysts, polyethylene, polyolefins, silica

## Abstract

Silica-supported chromium oxide catalysts, also named Phillips chromium catalysts (PCCs), provide more than half of the world’s production of high- and medium-density polyethylenes. PCCs are usually prepared in the Cr(VI)/SiO_2_ form, which is subjected to reductive activation. It has been explicitly proven that CO reduces Cr(VI) to Cr(II) species that initiate ethylene polymerization; ethylene activates Cr(VI) sites as well, but the nature of the catalytic species is complicated by the presence of the ethylene oxidation products. It is widely accepted that the catalytic species are of a Cr(III)–alkyl nature, but this common assumption faces the challenge of “extra” hydrogen: the formation of similar species under the action of even-electron reducing agents requires an additional H atom. Relatively recently, it was found that saturated hydrocarbons can also activate CrO_x_/SiO_2_, and alkyl fragments turn out to be bonded with a polyethylene chain. In recent years, there have been numerous experimental and theoretical studies of the structure and chemistry of PCCs at the different stages of preparation and activation. The use of modern spectral methods (such as extended X-ray absorption fine structure (EXAFS), X-ray absorption near-edge structure (XANES), and others); *operando* IR, UV–vis, EPR, and XAS spectroscopies; and theoretical approaches (DFT modeling, machine learning) clarified many essential aspects of the mechanisms of CrO_x_/SiO_2_ activation and catalytic behavior. Overall, the Cosse–Arlman mechanism of polymerization on Cr(III)–alkyl centers is confirmed in many works, but its theoretical support required the development of nontrivial and contentious mechanistic concepts of Cr(VI)/SiO_2_ or Cr(II)/SiO_2_ activation. On the other hand, conflicting experimental data continue to be obtained, and certain mechanistic concepts are being developed with the use of outdated models. Strictly speaking, the main question of what type of catalytic species, Cr(II), Cr(III), or Cr(IV), comes into polymerization still has not received an unambiguous answer. The role of the chemical nature of the support—through the prism of the nature, geometry, and distribution of the active sites—is also not clear in depth. In the present review, we endeavored to summarize and discuss the recent studies in the field of the preparation, activation, and action of PCCs, with a focus on existing contradictions in the interpretation of the experimental and theoretical results.

## 1. Introduction

Coordination polymerization of ethylene and α-olefins is used worldwide to produce most of the polyolefins, such as high-density polyethylene (HDPE), linear low-density polyethylene (LLDPE), isotactic polypropylene (PP), and others; the world’s annual polyolefin output exceeds 200 million tons [1]. Being discovered and commercialized in the mid-1950s, silica-supported chromium oxide catalysts, also called Phillips chromium catalysts (PCCs), are widely used in the production of polyethylenes [2]. Compared with Ziegler–Natta and single-site catalysts, PCCs demonstrate unique polymerization behavior with a formation of polyethylenes that have broad molecular weight distributions (MWDs) and polyethylene microstructures that include long-chain branched (LCB) fragments [3]. Polyethylenes, obtained using PCCs, are perfectly suited for blow-molding products like containers, fuel tanks, and pipes, occupying about half of the world’s HDPE market [3].

**Scheme 1:**
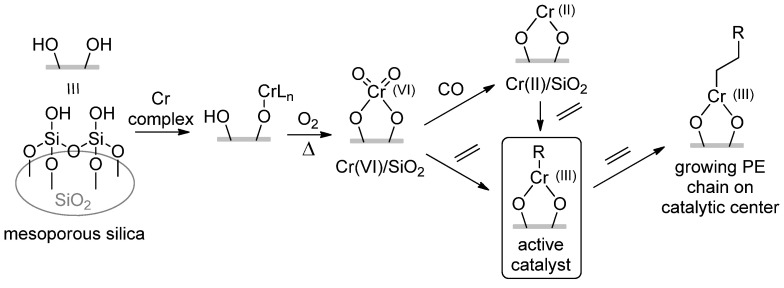
The generally accepted simplified mechanism of the formation and action of Phillips chromium catalysts (PCCs).

Compared with the great success in commercialization and industrial application, the scientific understanding of the processes of the formation, structure, and oxidation state of the active sites, as well as their initiation and polymerization mechanisms, is still incomplete and in many ways contradictory. These scientific problems have been discussed in a number of topical reviews [3,4,5,6,7,8,9,10,11,12,13,14]. The preparation and action of PCCs include the stages of impregnation of the Cr compounds on mesoporous silica, oxidative calcination with a formation of Cr(VI) species, reductive activation of Cr(VI) species, formation of the active catalyst, and polymerization. One of the possible reaction pathways, generally accepted to date, is presented in a simplified manner in Scheme 1. However, it should be noted that the hypothesis about the Cr(IV) and even Cr(II) nature of the catalytic species has not yet been refuted.

The main chemical problem of the reaction mechanism, presented in Scheme 1, is the interaction of the even-electron reducing agent, ethylene, with Cr(II) or Cr(VI) sites with a formation of Cr(III)-alkyl species. The formation of these species requires the participation of an additional H atom (the problem of “extra” hydrogen). To solve this problem, a number of complex and challenging reaction mechanisms have been proposed.

In this review, we tried to present and discuss the results of recent investigations on the preparation of chromium-impregnated supports and their thermooxidative treatment, reduction, and catalyst’s activation with a focus on mechanistic aspects of these processes.

## 2. Preparation of Chromium-Impregnated Supports

### 2.1. Type and Chemical Characteristics of the Support—The Preference for Silica

Over nearly 70 years of development of PCCs, various inorganic phases, including γ-Al_2_O_3_ and aluminum phosphates, have been studied as supports for chromium oxide catalysts, but other phases are inferior to silica in terms of activity of the catalysts obtained [3]. Chemical modification of the silica surface provides significant benefits over the use of inorganic phases different from silica. In particular, TiO_2_- and Al_2_O_3_-containing silica-based PCCs are long-lasting and have been successfully commercialized [8,15,16]. In further studies, fluorination of the silica supports at the stage of thermal treatment proved to be quite effective in terms of catalytic activity and PE characteristics [3]; the impact of fluorination of silica on the properties of PCCs was studied recently by Liu et al. [17,18] (see Section 6.5).

However, in any case, it is silica that is the support for industrially applicable PCCs (and, as is typical, other industrially important catalysts). It is therefore only natural that experimental and theoretical studies of silica, and especially mesoporous and amorphous silica, were conducted previously [19]. In the present work, we decided to confine ourselves to the discussion of basic and recent works in this field relevant to the subject of this review.

Since the first stage of the preparation of PCCs represents the treatment of mesoporous silica by a solution of Cr complex, the chemical nature of the silica surface, specifically the reactivity of the ≡Si–OH groups, cannot be a matter of indifference. The silica surface contains nonhydroxylated silica atoms (Q^4^) and three types of silanol groups: heminal =Si(OH)_2_ (Q^2^), vicinal =Si(OH)OSi(OH)= (Q^3^), and isolated ≡Si–OH (Q^3^). The bimodal acidity of the silica surface, which was proven experimentally in earlier studies [20], was confirmed by the results of recent theoretical investigations and is undoubtedly valuable for the development of the theoretical model of PCCs. In particular, Sulpizi et al. identified highly acidic convex heminal and vicinal silanol groups (pK_a_ = 2.9 and 2.1, respectively) as well as low-acidic concave geminals and isolated ≡Si–OH groups (pK_a_ = 8.9 and 10.3, respectively) [21]. In 2019, the scientific groups of Gierada and Tielens published a paper devoted to the study of the acidity of ≡Si–OH groups on the silica surface [22]; the results of their investigation refined some findings of previous works but were not applied to PCCs.

**Figure 1:**
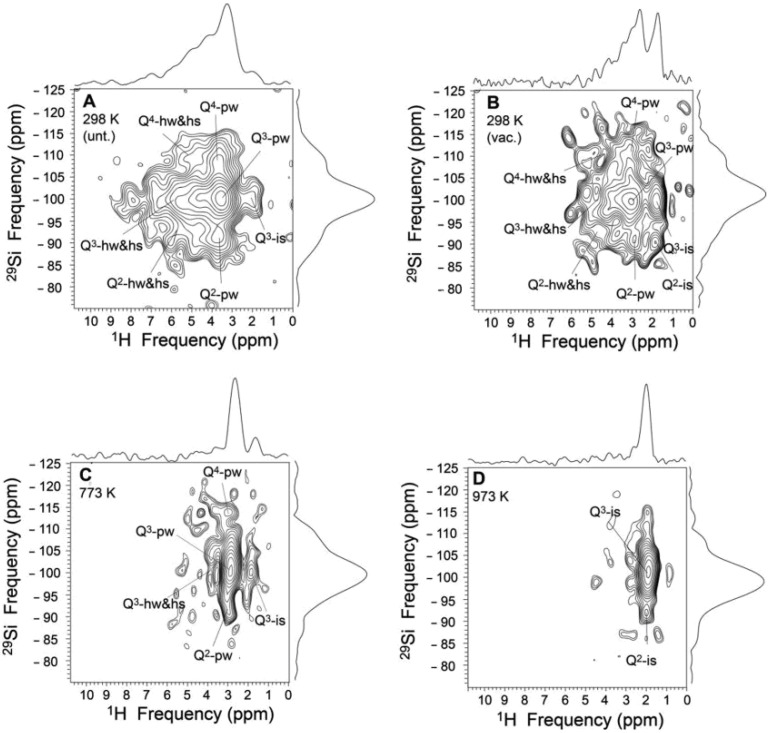
^1^H–^29^Si HetCor spectra and projections in ^1^H and ^29^Si dimensions at 9.4 T for 7 nm amorphous silica nanoparticles before and after dehydration at 20 Torr at 20 °C for 6 h (**A**,**B**, respectively) and after dehydration at 773 K (**C**) and 973 K (**D**) for 2 h. Contour lines are drawn with 15 lines in logarithmic scale with a spacing factor of 1.2. The labels is, hs, hw, and pw refer to isolated silanol, hydrogen-bonded silanol, hydrogen-bonded water, and physisorbed water, respectively. Reprinted with permission from [23]. Copyright (2013) Elsevier B. V.

Besides the interaction of hydrated (or noncalcined) silica with Cr sources, preliminarily calcined silica can be used in combination with solutions of Cr complexes in aprotic solvents. The surface dehydroxylation during the thermal pretreatment can affect the properties of silica; the changes in chemical nature of Si–OH groups during calcination were determined by Kim and Lee with the use of ^1^H–^29^Si solid-state HetCor NMR [23] (Figure 1). Also note that the calcination of amorphous Aerosil^®^ at 700 °C takes place without significant loss of specific surface area, and the remaining statistical population of isolated ≡Si–OH groups has a coverage of ~0.8 OH∙nm^−2^ [24].

**Figure 2:**
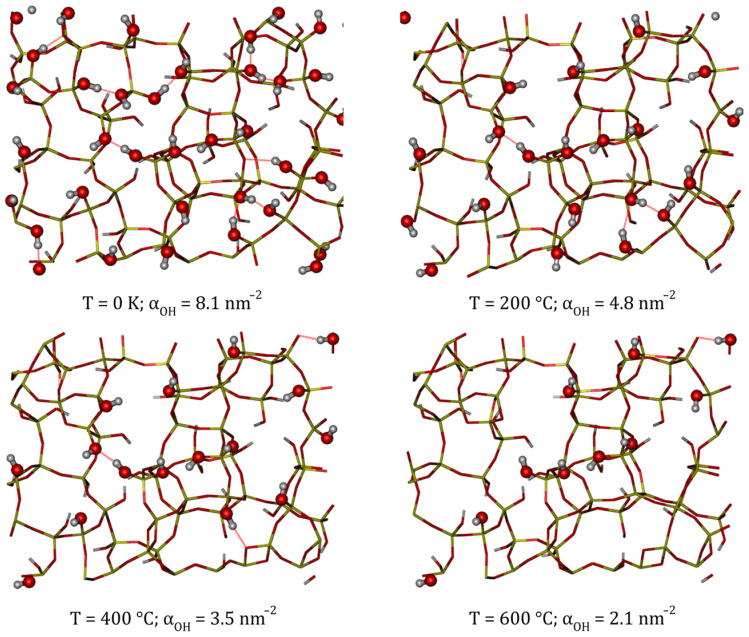
Representative images of amorphous silica surface models at various temperatures. Reprinted with permission from [25]. Copyright (2014) American Chemical Society.

With the use of a small model containing 456 atoms (using a supercell with dimensions of 23.742 Å × 18.276 Å × 17.404 Å), Johnson and colleagues developed a model of the SiO_2_ surface that can faithfully represent the physics and chemistry of real surfaces, which was directly confirmed by macroscopic measurements of the silanol number and type as a function of temperature [25]. As can be seen in Figure 2, the thermal treatment of the amorphous silica results in the formation of ≡Si–OH containing islets. It can be assumed that these local ≡Si–OH concentrations are perfect templates for the coordination of both chromates and dichromates (see Section 3), but this aspect was not noticed in later works.

Two years later, Comas-Vives suggested a model of SiO_2_ on the basis of a bulk cubic unit with a lattice parameter of 21.39 Å and containing 216 SiO_2_ units (648 atoms) [26]. The fundamental difference of the Comas-Vives model from the models proposed previously is an assumption of SiO_2_ reconstruction during thermal dehydration, allowing it to reach a ≡Si–OH coverage of 1.1 OH∙nm^−2^ with an average energetic cost of 143 kJ∙mol^−1^ per water molecule. This energetics is significantly more favored than the one (262 kJ∙mol^−1^ per water molecule for the coverage of 1.5 OH∙nm^−2^) previously reported by Ugliengo et al. for their periodic amorphous model [27] and allows the substantiation of the experimental value of the ≡Si–OH coverage (0.8 OH∙nm^−2^) obtained for a silica surface treated in vacuo at 700 °C. Dehydration results in a formation of the surface containing strained Si–O–Si fragments, which cannot but affect the properties of the supported catalyst.

**Figure 3:**
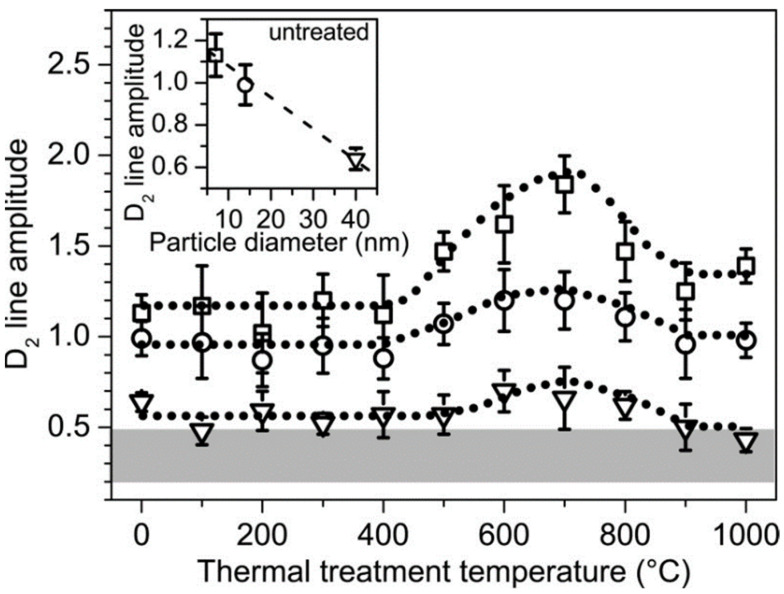
D_2_ line amplitude in the materials (☐) AE380, (O) AE150, and (▽) AEOX50, estimated from the normalized Raman spectra, as a function of the thermal treatment temperature. The gray stripe represents the D_2_ line amplitude variability observed in a lot of bulk silica materials of different commercial origins. The inset shows the D_2_ line amplitude in the untreated samples as a function of the mean diameter of the primary particles. Reprinted with permission from [28]. Copyright (2010) American Chemical Society.

The formation of strained siloxane fragments during calcination was proven experimentally by Raman spectroscopy [28]: the spectral D_2_ line that corresponds to strained (SiO)_3_ fragments had a maximum at ~700 °C (Figure 3).

**Figure 4:**
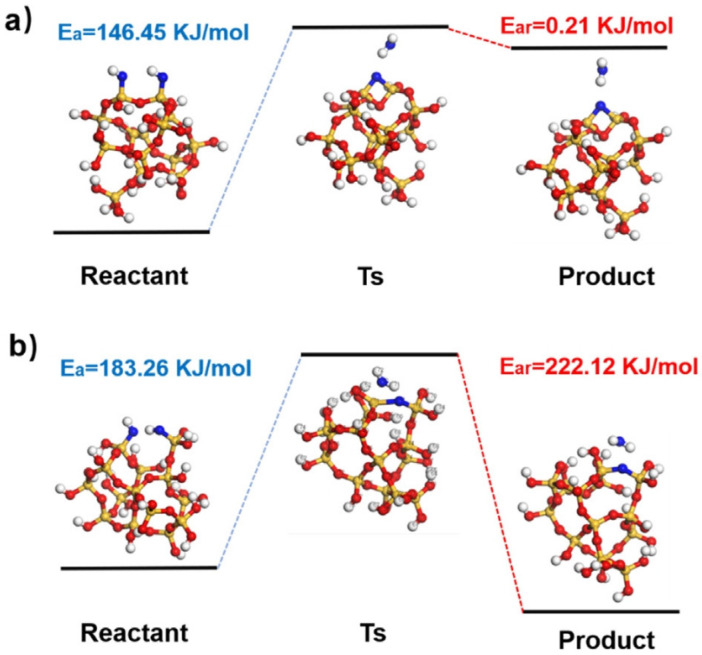
Reaction process diagrams of E_a_ (dehydration) and E_ar_ (rehydration) for (**a**) Si–OH^C^ and (**b**) and Si–OH^S^. Colored atoms: H, white; Si, yellow; O, red; O, involved in dehydration, blue. Reprinted with permission from [29]. Copyright (2020) Elsevier B. V.

In 2020, Cheng et al. quantitatively analyzed the changes in the number of various hydroxyl groups after dehydroxylation and rehydration of silica using TGA, ^29^Si MAS NMR, and DFT modeling [29]. They proposed two types of hydroxyl groups depending on the relative positions of them: chain-adjacent hydroxyl groups (Si–OH^C^) and spatially adjacent hydroxyl groups (Si–OH^S^) (Figure 4). Dehydroxylation of two Si–OH^C^ formed a four-atom ring structure, and the resulting O–Si–O bond angle deviation from an optimal value was up to 19.56°. The Si–OH^S^ dehydroxylation formed a more stable ring but required higher temperatures (higher E_a_, Figure 4). These results seem to match up with the formation of vicinal Si–OH islets in Ewing’s model [25].

### 2.2. Porosity of the Support

As was shown by McDaniel, the molecular weight of PE decreased with a rising pore diameter up to about 200 Å, at which point MW became relatively constant—in other words, polymer chains become longer when they are produced under the most restricted and crowded conditions [30]. From the mechanistic point of view, a proposed possible explanation was that in such crowded pores, polymer chains are inhibited from assuming the agostic β-H coordination that is a precursor for chain termination. From our side, we note that the mechanistic alternative to β-H elimination, namely, β-H transfer to ethylene, requires even more space.

On the other hand, a larger pore volume and pore diameter are required for the disintegration of the catalyst particles during polymerization. This ensures high and consistent activity as well as excellent morphology of the PE [16]. However, the pronounced dependence of the PE characteristics from support properties allowed the development of a simple and efficient method of the modification of PCCs based on the treatment of commercial mesoporous silica by Si(OEt)_4_ oligomers and the Cr source before oxidative calcination [31]. As a result, a less-than-two-fold reduction in activity was coupled with a formation of PE with higher LCB content and an order of magnitude lower melt index (MI) and high-load melt index (HLMI) values, determined according to ASTM D1238 [32] using 2.16 and 21.6 kg loads, respectively, with close molecular weight characteristics.

### 2.3. Chromium Source

**Figure 5:**
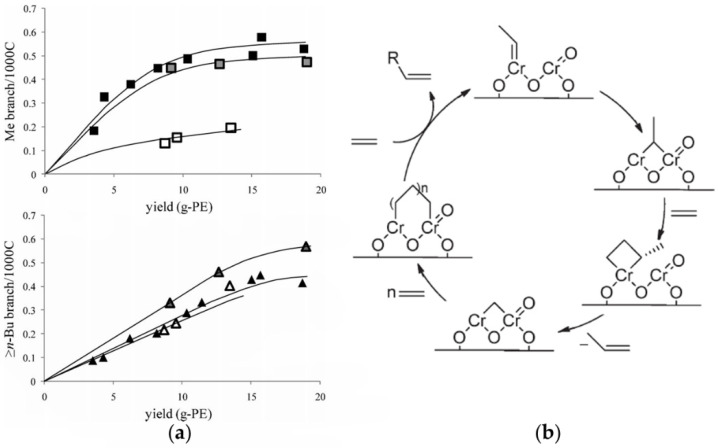
(**a**) Influences of the chromium nuclearity on Me branching (**top**) and ≥*n*-Bu branching (**bottom**), black: industrial Phillips catalyst, white: Cr(η^3^-allyl)_3_-based catalyst, gray: Cr_2_(η^3^-allyl)_4_-based catalyst; (**b**) plausible mechanism for enhancement of propylene formation on dinuclear chromium species. Reprinted with permission from [33]. Copyright (2011) Wiley-VCH Verlag GmbH & Co.

In many experimental works and a recent tutorial review [3], McDaniel pointed out that the chromium source is not crucial for the preparation of PCCs because of the formation of silyl chromates (VI) during thermooxidative treatment and the mobility of the Cr (VI) species at high temperatures. However, in 2011, Terano and colleagues reported the results of a comparative study of PCCs containing mononuclear and dinuclear Cr species, where nuclearity was set by the nuclearity of the Cr sources Cr(η^3^-allyl)_3_ and Cr_2_(η^3^-allyl)_4_, respectively [33]. After impregnation, hydrogenation, and thermooxidative treatment at 600 °C, the catalysts demonstrated qualitatively different reactivities in homopolymerization of ethylene: an increased formation of Me branches was detected when using the Cr_2_(η^3^-allyl)_4_-based catalyst, and the contents of *n*-Bu (and longer) branches were close (Figure 5a). To explain this fact, Terano and colleagues proposed a new metathesis mechanism of the formation of propylene with the participation of binuclear catalytic species (Figure 5b). 

One would assume that, despite Cr(VI) mobility, impregnation of Cr complexes on the surface of calcined silica may result in the partial retention of the cluster structure (in view of the islet nature of the calcined silica surface [25]). In that case, the studies of trinuclear basic carboxylates of Cr(III), tetranuclear [(EH)_2_CrOH]_4_ [34], and other hydrocarbon-soluble Cr clusters are not meaningless, in view of the rich coordination chemistry of Cr.

Another prospective aspect of the preparation of the support and impregnation of the Cr complexes may be related to the structure of the amorphous silica. Although the generally accepted view on the impregnation mechanism regards it as a reaction of ≡Si–OH with a formation of ≡Si–O–Cr species, the involvement of highly strained Si–O–Si fragments of calcined silica to the chemisorption of the chromium does not seem impossible.

## 3. Thermooxidative Treatment of Cr-Impregnated Supports

### 3.1. Experimental Observations

During thermooxidative treatment, Cr sites are oxidized to Cr(VI) species and evenly distributed on the silica surface [35]. In a recent feature article [36], McDaniel showed that the oxidative calcination process directly affects the catalytic activity of the PCCs and influences the concentration and diversity of the active sites. PE characteristics (polymer MW, the shape and breadth of the MWD, the efficiency of comonomer and macromer incorporation, and the location of both short-chain branches (SCBs) and LCBs within the MW distribution) depend on the nature of the catalytic centers. The review [36] summarizes the experimental observations of the activity of PCCs as a function of the calcination temperature; the dependence of the properties of Cr(VI)/SiO_2_ and PE characteristics on the calcination temperature is presented in the Table 1.

**Figure 6:**
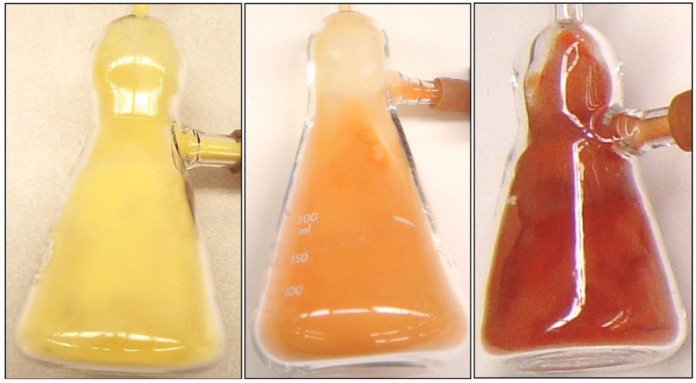
Cr(VI)/silica catalysts change color with increasing silica dehydroxylation, from yellow to orange and finally to red. Reprinted with permission from [36]. Copyright (2017) Elsevier B. V.

Besides activity, calcination temperature directly affects the look of the Cr(VI)/SiO_2_ (Figure 6). The change in color is conventionally attributed to the formation of mono-, di-, and polychromates—since the increase in the temperature results in a decrease in the concentration of ≡Si–OH groups, and dichromate can tolerate wider silanol spacing; the formation of di- and polychromates was confirmed experimentally, in particular with the use of diffuse reflectance infrared Fourier transform spectroscopy (DRIFTS) and diffuse reflectance UV–vis spectroscopy (DRS) [37]. “More colored” oxidized PCCs often form more active polymerization catalysts; however, in some other works (e.g., [38]), Cr clusters are seen as inactive.

An important feature of the thermooxidative treatment is the mobility of Cr(VI), which results in even distribution of Cr on the support surface [35]; the nature of the mono-Cr sites traditionally seemed to be from the distribution of (≡SiO)_2_CrO_2_ and (≡SiO)_4_CrO species [39,40,41]. However, in 2018, Peek and colleagues reinvestigated the electronic structure of isolated Cr(VI)/SiO_2_ sites using multiple electronic spectroscopies of transparent xerogel monoliths [42]. In the UV–vis spectrum, they detected the presence of previously reported peaks at 22,800, 29,100, and 41,500 cm^−1^ and a previously unresolved band at ~36,900 cm^−1^. The emission with λ_max_ = 13,600 cm^−1^, emanating from the lowest excited state, was also detected. Assignment of the excited states was performed using time-dependent density functional theory (TD-DFT) calculations (see below), and as a result, all the observed electronic transitions were attributed to the presence of (≡SiO)_2_CrO_2_ sites, with no evidence for a significant contribution from (≡SiO)_4_CrO sites.

### 3.2. Theoretical Studies

In 2013, Handzlik and colleagues reported the results of comprehensive periodic and cluster DFT investigations of the monomeric Cr(VI)/SiO_2_ species under dehydrated conditions using various advanced models of the SiO_2_ surface, based on the β-cristobalite structure and different amorphous model structures [39]. All the approaches led to the same general conclusion that the (≡SiO)_2_CrO_2_ dioxo species are more stable than the (≡SiO)_4_CrO monooxo species. On the other hand, the calculated preference for the (≡SiO)_2_CrO_2_ species was stronger in the case of the β-cristobalite-based models than for the amorphous surface models due to the higher flexibility of the latter. As a result of these theoretical studies, a comprehensive background for further theoretical simulations of the reduction and activation of the Cr catalytic species has been established.

**Figure 7:**
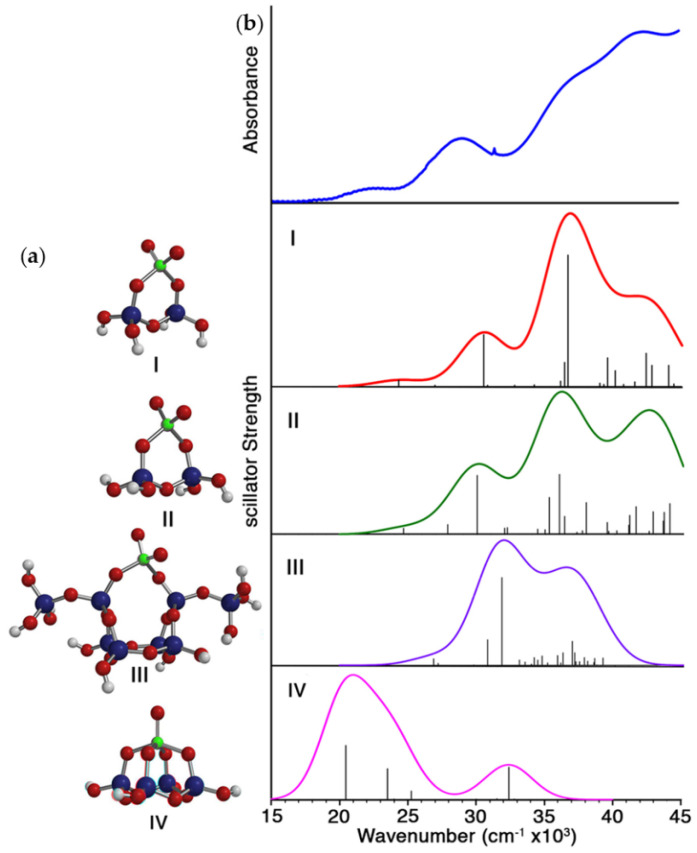
(**a**) Structures of cluster models used in the calculations. The color scheme: green Cr, red O, blue Si, and gray H. (**b**) Comparison of electronic absorption spectra: (top) experimental spectrum for Cr(VI)/SiO_2_ and simulated spectra based on TD-DFT calculations for clusters I–IV. Vertical lines show the energies of the one-electron promotions that comprise the transitions. The half-width of each band at half-height was set to 4000 cm^−1^ in the simulations. Reprinted with permission from [42]. Copyright (2018) American Chemical Society.

To assign the bands in UV–vis absorption and luminescence spectra, Peek et al. performed TD-DFT calculations on three cluster models for (≡SiO)_2_CrO_2_ sites and one cluster model for a (≡SiO)_4_CrO site (Figure 7a) [42]. As can be seen in Figure 7b, the real spectral view of the Cr(VI)/SiO_2_ sample can be a superposition of the (≡Si–O)_2_CrO_2_ sites in different ligand environments, whereas there were no spectroscopic signs of the (≡SiO)_4_CrO species in the sample under study.

**Figure 8:**
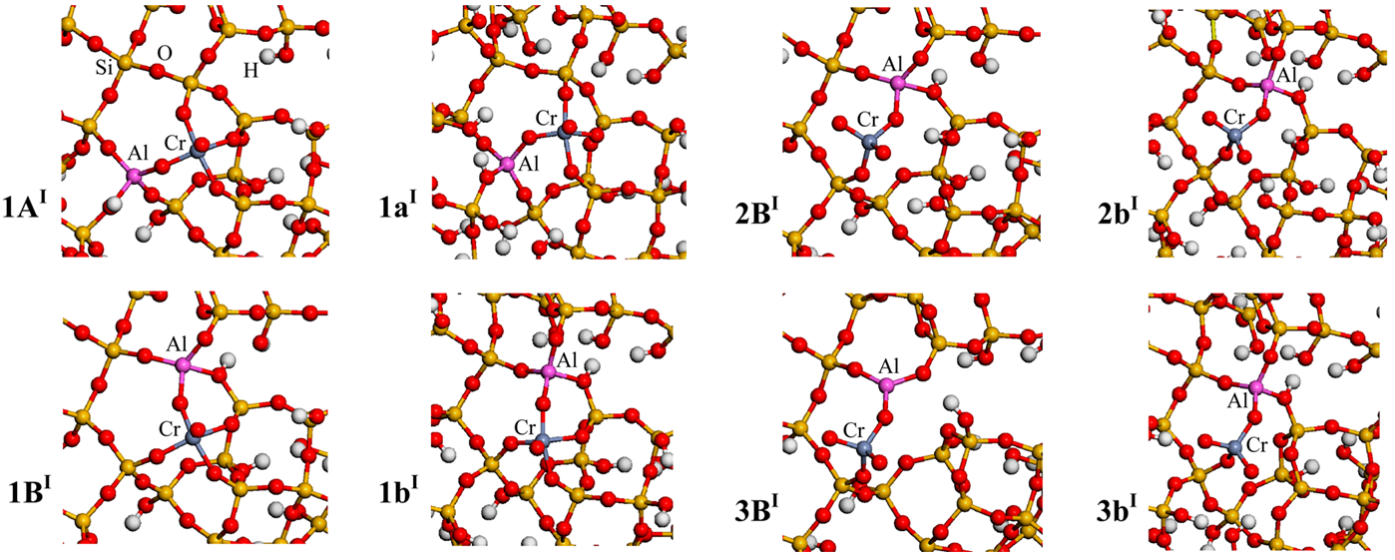
Optimized structures of the monooxo (**1A^I^**,**1a^I^**,**1B^I^**,**1b^I^**) and dioxo (**2B^I^**,**2b^I^**,**3B^I^**,**3b^I^**) Cr(VI) species on Al-modified silica, represented by the periodic (**1A^I^**,**1B^I^**–**3B^I^**) and corresponding cluster (**1a^I^**,**1b^I^**–**3b^I^**) models. One Al site per Cr site is assumed. Reprinted with permission from [43]. Copyright (2016) American Chemical Society.

Very interesting results were obtained by the same scientific group during the studies of a Cr(VI)/Al_2_O_3_/SiO_2_ system [43] using periodic and cluster models of silica developed earlier [39], with a replacement of ≡Si–O–Cr fragments by =Al–O–Cr or ≡Al⋯O–Cr fragments. The example of the hydroxylated set of the optimized structures with one Al–O–Cr fragment is presented in Figure 8, and this is just a small part of the calculations that have been performed. The relative stability of dioxo and monooxo species and the thermodynamics of dehydration were estimated. The results of the modeling allowed the explanation of the known experimental fact of a higher ratio of the monooxo to dioxo surface Cr(VI) species for Cr(VI)/AlOx/SiO_2_ compared to the Cr(VI)/SiO_2_ system. This difference was attributed to the difference in the ligand environments and geometry: a monooxo Cr(VI) species located in the vicinity of two Al sites usually adopts a pseudotetrahedral geometry, in contrast to square pyramidal monooxo Cr(VI) species on silica.

Populations of different ≡Si–OH groups on the silica surface can be estimated using spectral methods, in particular, the ^29^Si cross-polarization magic angle spin (CP MAS) and ^1^H MAS NMR techniques. As was shown by Cheng et al. [44] in experiments on calcination of SiO_2_ and Cr(VI)/SiO_2_ at 120–800 °C, for the Cr(VI)/SiO_2_ treated at temperatures lower than 300 °C, the amount of residual silanol groups was much lower than that of SiO_2_. The geminal silanols almost disappeared in Cr(VI)/SiO_2_ at 120 °C but were detected at 300 °C in SiO_2_. With a further increase in temperature, the amount of single silanols was slower to decrease for the Cr(VI)/SiO_2_ samples, thus indicating that the presence of the grafted chromate species stabilizes residual single silanols that can be involved in the activation of Cr(VI)/SiO_2_ by ethylene (see Section 4).

**Scheme 2:**
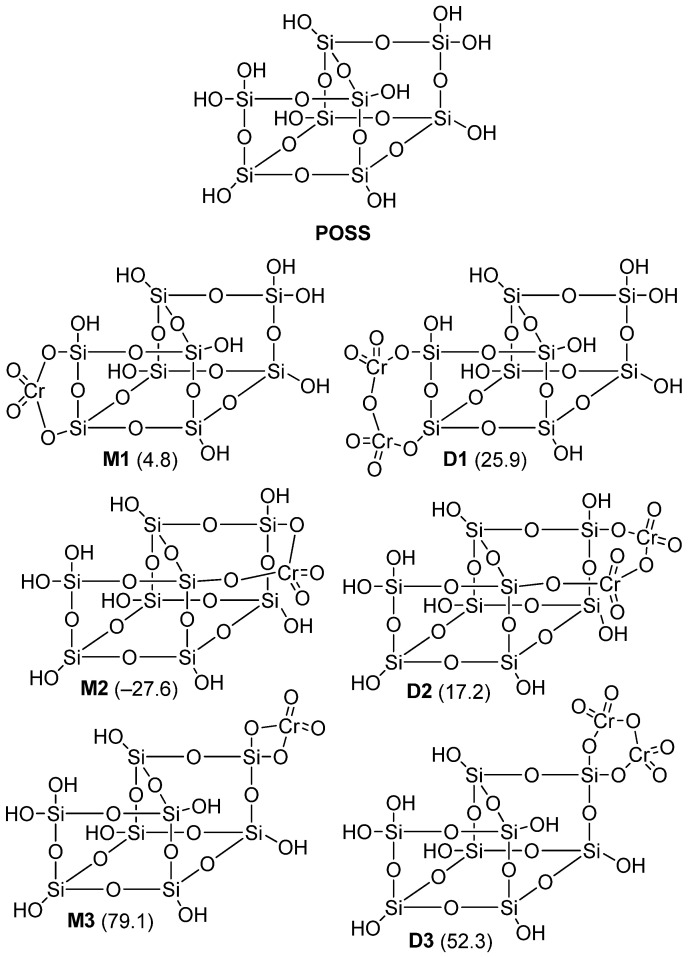
Polyoligomericsilsesquioxane (POSS) model and computational models for Cr(VI) ester on six kinds of silanol pairs on POSS; the enthalpies of the formation of Cr (VI) esters (kJ∙mol^−1^) are given in parentheses [44].

DFT modeling with the use of a simple, well-defined polyoligomeric silsesquioxane (POSS) model showed that the combination of geminal and single silanols is the most thermodynamically favored for CrO_3_ grafting (**M2** in Scheme 2). The model is extremely simplified but fairly visual; in addition, the more complex Cr(VI)/SiO_2_ models of Handzlik and colleagues [39], strictly speaking, do not consider the pathways of chromate formation.

However, the relevance of the POSS model for the explanation of the results of real experiments on ethylene polymerization using PCCs seems doubtful in view of the experimental studies of POSS-impregnated Cr complexes as a model for PCCs [45]. These complexes were found to be an order of magnitude less active in comparison with Cr/SiO_2_ systems.

**Figure 9:**
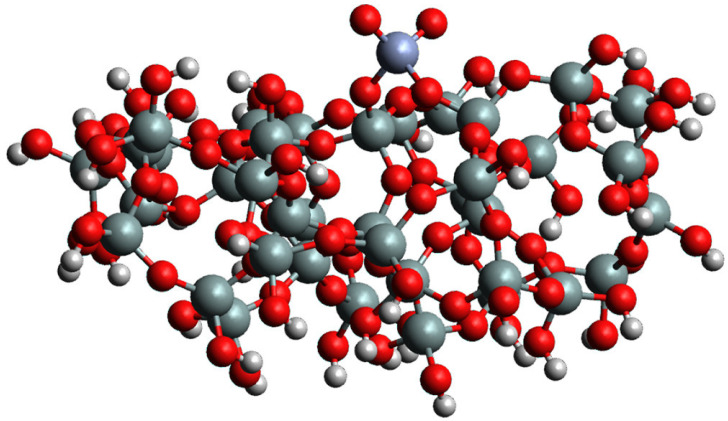
Example of an atomistic Cr-doped silica cluster. The color scheme: blue Cr, red O, gray Si, and white H. Reprinted with permission from [46]. Copyright (2021) American Chemical Society.

To conclude this section, the recent study of Peters and colleagues [46] should be mentioned. They noted that in addition to the quenched disorder (locked in by strong bonds that do not rearrange on the time scale of the catalytic reactions) and dynamical disorder (the hydrogen bond network of the silanol groups that can rearrange quickly during the reactions), the procedural disorder, induced by the computational protocol, does matter. In their work, a detailed investigation of the procedural disorder by comparing two geometry optimization protocols for the grafting of CrO_3_ on the silica surface was presented. The first protocol started from a silica structure obtained from classical molecular dynamics (MD) simulations, and only the central region surrounding the metal was relaxed (MD-Opt). The second protocol (QM-Opt) relaxed a large portion of the cluster at the QM level before grafting. The comparison of these protocols using an empirical disordered lattice model allowed them to explore significantly larger cluster model sizes and atomistic models treated at the QM level (Figure 9, as an example) and showed that the QM-Opt protocol is more computationally efficient than the MD-Opt protocol when the size of the relaxed zone is sufficiently large to provide an acceptably small amount of procedural disorder. The results of the work, devoted to the Cr(VI)/SiO_2_ species, clearly point to the doubtful validity of the chromasiloxane structures still used in the DFT modeling of the catalytic species (see Section 4, Section 5 and Section 6).

## 4. Reduction and Activation of the Cr(VI)/SiO_2_

### 4.1. Reduction by CO

The reaction of Cr(VI)/SiO_2_ with CO at 350 °C quantitatively leads to the formation of coordinatively unsaturated Cr(II) species that are quite active for ethylene polymerization [5]. It was previously thought that Cr(II)/SiO_2_ species formed by this method are similar to Cr(II)/SiO_2_ species formed during the reaction of Cr(VI)/SiO_2_ with ethylene; however, as was discussed in Section 4.1, the products of the latter reaction (HCOOMe, etc.) are able to achieve complexation with the Cr(II) centers, unlike CO_2_. Hence, the process of the reduction of Cr(VI)/SiO_2_ by CO is simpler in comparison with the reaction with ethylene. Also note that the reaction of Cr(VI)/SiO_2_ with ethylene can lead to the formation of Cr(II)/SiO_2_ immediately, whereas the reaction with CO can proceed in a stepwise manner. When using an optically transparent monolith of Cr(VI)/mesoporous SiO_2_, a very clean, stepwise reduction by CO of Cr(VI) first to Cr(IV) and then to Cr(II) was detected using in situ UV–vis spectroscopy [47]. Both the intermediate Cr(IV) and final Cr(II) states showed XANES consistent with these oxidation state assignments. The intermediate Cr(IV) sites were inactive toward ethylene at 80 °C.

**Figure 10:**
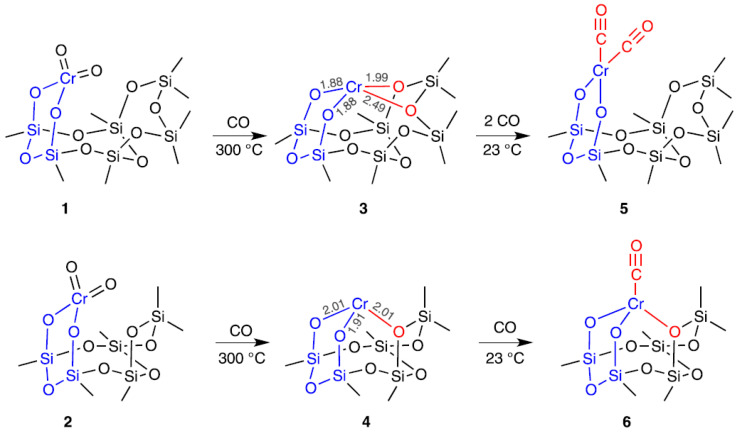
Proposed structures for two different chromate sites (**1**,**2**) embedded in 6-membered chromasiloxane rings (blue) on highly dehydroxylated amorphous silicas and the corresponding Cr(II) sites (**3**,**4**) formed upon reduction (showing the distances obtained by EXAFS curve fitting), as well as their corresponding carbonyl complexes (**5**,**6**). Additional siloxane and carbonyl ligands are shown in red. Reprinted with permission from [48]. Copyright (2012) Elsevier B. V.

A study of the coordination environment of the Cr(II) sites, obtained by the reduction of Cr(VI)/SiO_2_ samples prepared at 500 and 800 °C, was conducted by Zhong and colleagues in 2012 [48]. The EXAFS showed that (SiO)_2_Cr(II) sites are coordinated by either two or one additional siloxane ligands depending on the temperature of the thermooxidative treatment of CrO_x_/SiO_2_ (500 or 800 °C, respectively) and thereby demonstrated different reactivity toward both CO and ethylene (Figure 10). The latter three-oordinated sites showed higher activity in the polymerization of ethylene.

**Figure 11:**
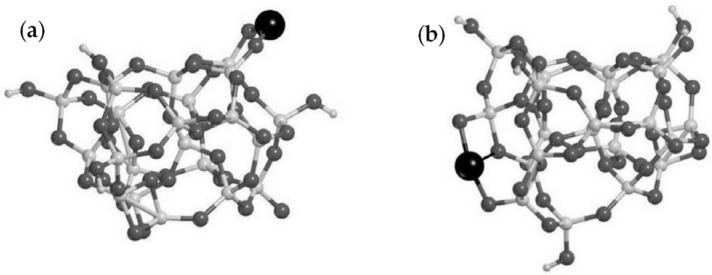
Graphical representation of Cr_A_(II) (**a**) and Cr_B_(II) (**b**) clusters adopted to model a highly dehydroxylated Cr(II)/SiO_2_ system. Small white spheres: H atoms. Big white spheres: Si atoms. Gray spheres: O atoms. Black spheres: Cr atom. Reprinted with permission from [49]. Copyright (2015) Elsevier B. V.

Experimental and theoretical studies of Cr(II) sites on highly dehydroxylated silica were conducted by Groppo and colleagues in 2015 [49]. The samples of PCCs (0.1 wt% of Cr) were calcined at 550, 650, and 750 °C under dynamic vacuum and in the presence of O_2_ and then reduced by CO. Surprisingly, the highest catalytic activity was demonstrated by the sample prepared at 550 °C. UV–vis and FT-IR spectral studies confirmed the presence of two types of Cr(II) sites with different degrees of coordinative unsaturation [5]: Cr_A_(II) sites that are able to coordinate up to two CO molecules at room temperature and Cr_B_(II) sites that form monocarbonyl adducts. For both types of site, a theoretical model, based on H_6_O_48_Si_22_Cr clusters (Figure 11), was developed. A good agreement between calculated and experimental UV–vis and FT-IR spectral data was demonstrated, but the model was not used for simulations of the reactions of Cr(II)/SiO_2_ with ethylene.

**Figure 12:**
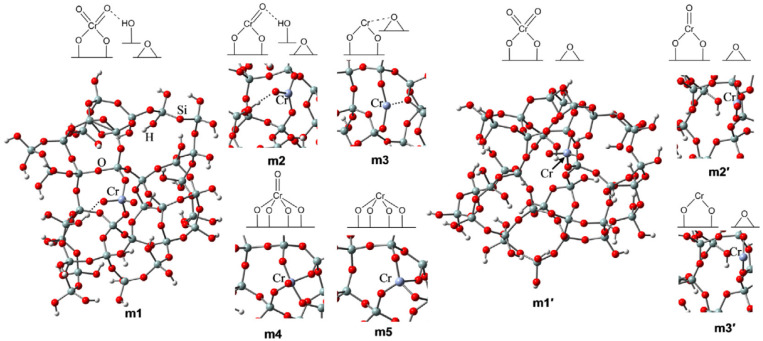
Optimized structures of the monomeric dioxo (**m1**,**m1′**) and monooxo (**m4**) Cr(VI) species and the corresponding Cr(IV) (**m2**,**m2′**,**m5**) and Cr(II) (**m3**,**m3′**) species on silica (cluster models). For clarity, only a fragment of the silica cluster is shown in most cases. Reprinted with permission from [50]. Copyright (2016) Elsevier B. V.

Based on the results of the modeling of Cr(VI)/SiO_2_ [39], Gierada et al. performed combined DFT and in situ UV–vis DRS investigations of the reduction of Cr(VI)/SiO_2_ by CO and H_2_ [50]. An extensive variety of monomeric Cr_1_ (Figure 12) and many more dimeric Cr_2_ species were optimized using cluster models.

**Scheme 3:**
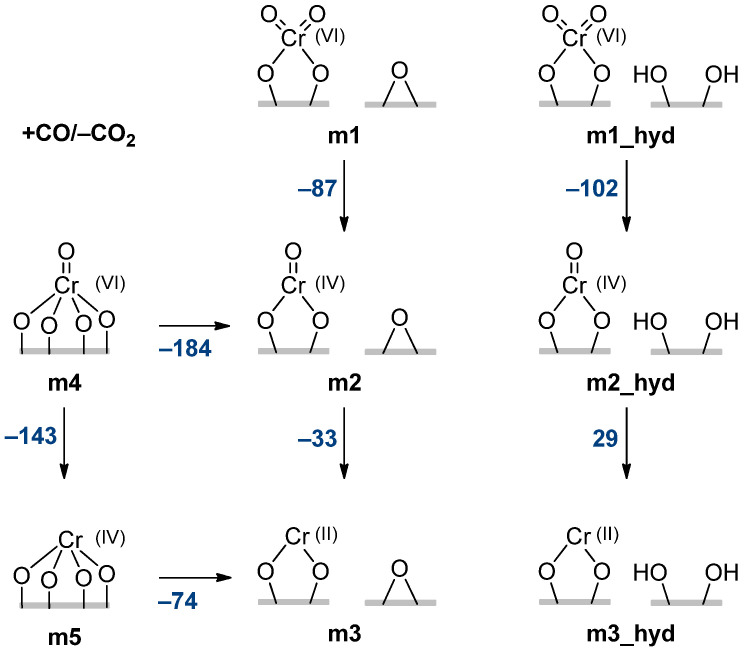
Calculated Gibbs energies at T = 873 K (ΔG_873_, kJ∙mol^−1^) for reduction reactions of the monomeric Cr oxide species on silica (cluster models) [50].

A two-step reduction of Cr_1_ species by CO with the reaction Gibbs energy for each step at T = 873 K is presented in Scheme 3. The reduction of the initial dioxo and monooxo Cr (VI) species to the corresponding Cr(IV) species was found to be a clearly exothermic and exergonic process; this effect was stronger for the monooxo Cr(VI) species. The thermodynamic driving force for the Cr(VI) → Cr(IV) reduction was greater than that for the Cr(IV) → Cr(II) reduction. In the case of the more probable monooxo Cr(IV) species, their reduction was predicted to be an exergonic or endergonic process, depending on the surface model applied (**m2** or **m2_hyd**). The authors of [50] also noted that the reduction with H_2_ must be a more endergonic and endothermic process than reduction with CO, by ~7 (ΔG_873_) and ~29 (ΔE) kJ∙mol^−1^, respectively. Therefore, it is even more likely that the reduction of Cr(IV) to Cr(II) may be an equilibrium or an endergonic process if H_2_ is used as the reducing agent. Another significant aspect of the use of H_2_ is a formation of H_2_O that can hydrolyze Si–O–Cr fragments with the subsequent formation of Cr_2_O_3_.

Similar, yet more intricate, calculations were conducted for numerous Cr_2_ species. The initial Cr(VI) dimeric species can be classified as double-dioxo, monooxo–dioxo, and double-monooxo species; the dimeric dioxo species were predicted to be the most stable. Optimization of the reduction products showed that the reaction of Cr_2_ species with CO is exothermic for almost all steps. However, in the case of Cr_2_ species, the intermediate formation of Cr(III) complexes was found to be possible.

**Figure 13:**
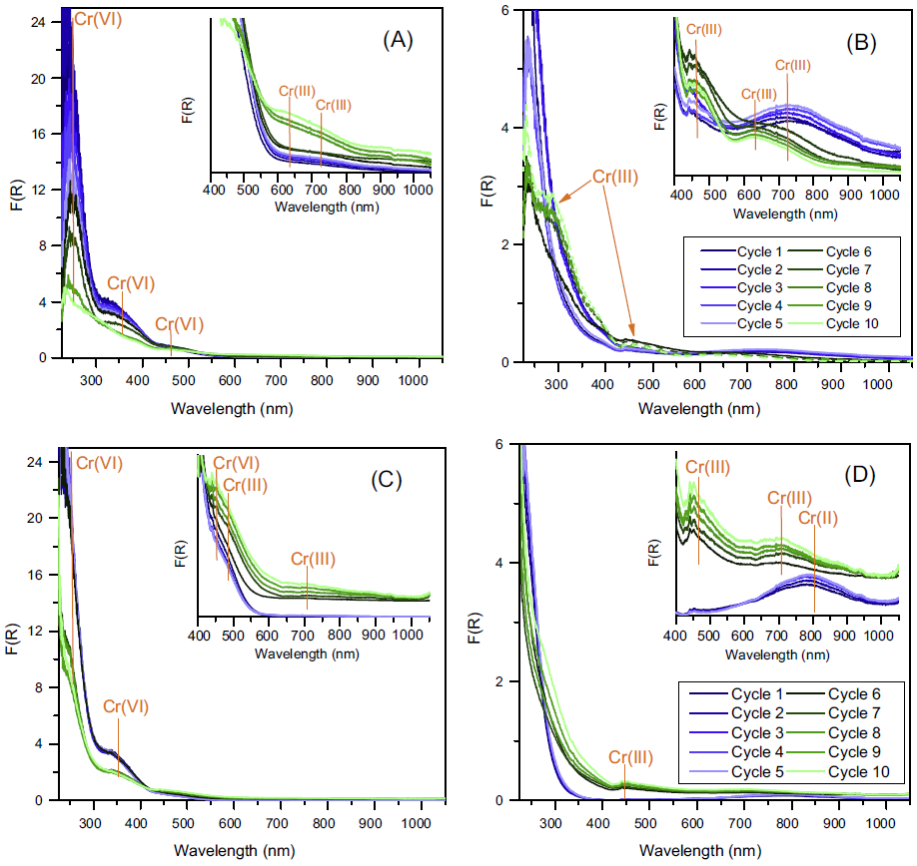
UV–vis DR spectra of a CrOx/SBA-1 sample recorded during consecutive reduction/oxidation cycles in the presence (green lines) and absence (blue lines) of water at 873 K. (**A**,**C**) Spectra of the sample oxidized with air; (**B**,**D**) spectra of the samples reduced with H_2_ and CO, respectively. Reprinted with permission from [50]. Copyright (2016) Elsevier B. V.

The behavior of CrO_x_/SBA-1 during its reduction with H_2_ or CO was studied in the presence and in the absence of water. In each experiment, the fresh CrO_x_/SBA-1 sample was first reduced by dry H_2_ or CO and then reoxidized by dry air at 600 °C. After five reduction/oxidation cycles, the next five cycles were carried out in the presence of H_2_O. The results of the study (Figure 13) largely confirm the theoretical findings. 

In particular, the reduction with dry H_2_ led to the formation of both Cr(III) and Cr(II) species. In each dry reduction cycle, a clear, broad d–d band appeared, indicating the simultaneous formation of Cr(III) and Cr(II) species. Reoxidation with dry air during the regeneration step restored the Cr(VI) species (characteristic bands at 270, 360, and 450 nm), but the intensity of these bands decreased, indicating only partial regeneration of Cr(VI). This effect was even clearer in the presence of H_2_O; moreover, in the cycles with wet H_2_ reduction, new bands at 290, 460, and 620 nm appeared, suggesting the formation of Cr_2_O_3_ particles. When using CO in the absence of water, Cr(II) species were formed almost selectively, and during the reoxidation step, the characteristic CT bands were restored completely (Figure 13C,D). In contrast, during wet reduction with CO, the band at 810 nm, characteristic of pseudo-octahedral Cr(II) species, shifted to a lower wavelength (ca. 720 nm). This shifting, together with the appearance of a new weak band at ~465 nm, confirmed that water suppresses the deep reduction to Cr(II). In the presence of wet CO, the reduction process led to the formation of Cr(III) species, but Cr_2_O_3_ particles were not detected. In this way, the lower efficiency of H_2_ as an activator for Cr(VI)/SiO_2_ received further experimental and first theoretical evidence.

**Figure 14:**
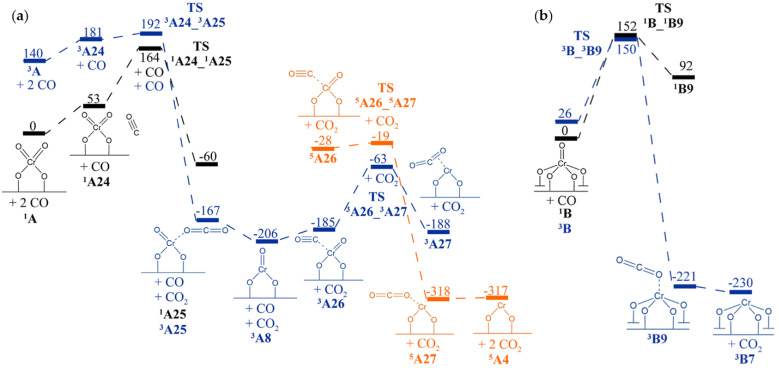
Gibbs energy profiles (kJ∙mol^−1^) at T = 373 K for the two-step reduction of the (**a**) dioxo and (**b**) monooxo Cr(VI) species with CO. Black, blue, and orange colors denote singlet, triplet, and quintet reaction pathways, respectively. Reprinted with permission from [41]. Copyright (2018) Elsevier B. V.

However, the kinetics of the reduction of Cr(VI) by CO is no less important than its thermodynamics. In a first theoretical study of the kinetics of this process [38], Gierada and Handzlik reported the results of DFT modeling of the interaction of dioxo and monooxo Cr(VI) clusters (Figure 14) with CO [41]. For dioxo species, the calculated ΔG^≠^ value (164 kJ∙mol^−1^) was higher than in the case of the reduction with ethylene that correlated with the higher temperatures needed for PCC activation by CO. In high contrast with a reaction with ethylene, which is a bad reducing agent for monooxo Cr(VI) species, reduction with CO had relatively low ΔG^≠^ (152 kJ∙mol^−1^), but the Cr(IV) product of the reduction proved to be stable.

In 2019, Mino and colleagues studied the photoinduced reduction of Cr(VI)/SiO_2_ by CO at room temperature [51]. The photoreduction by CO was less efficient than the thermal reduction at 350 °C; however, the relative amount of highly uncoordinated Cr(II) sites capable of forming multicarbonyls in the presence of CO at room temperature was much higher when the Cr(II) sites were obtained by photoreduction.

### 4.2. Reduction and Activation by Ethylene 

The conventional mechanism of the reduction of Cr(VI)/SiO_2_ involves the formation of Cr(II) species (Scheme 1), and formaldehyde has been long claimed as a product of ethylene oxidation [8,14]. In many works, Cr(II) species were viewed as “naked”, extremely low-coordinated Cr sites, covalently bonded to two or three ≡Si–O fragments [3,4,5,6,7,8]. Also note that in the previous work of Groppo and colleagues [52], the formation of two populations of Cr(II) sites with different catalytic activities was proposed.

In the early theoretical work of Liu and colleagues, for the simplest O(SiH_2_O)_2_Cr model, it was shown that the coordination of one CH_2_O molecule favors ethylene dimerization to but-1-ene through a Cr(IV)-hydride intermediate and metathesis to form propylene/ethylene through a chromacyclobutane intermediate [53]. 

The lack of CH_2_O detection by online mass spectrometry (MS) stimulated Barzan and colleagues to follow the activation experiment by *operando* FT-IR spectroscopy, aimed at directly detecting possible oxygenated species adsorbed at the catalyst surface [54]. Methyl formate HCOOMe was found to be the most probable oxygenated by-product formed via Tischenko reaction (but the formation of adsorbed formate species and methoxy groups grafted at the silica surface also cannot be excluded). In this way, the product of the reduction of Cr(VI)/SiO_2_ by ethylene represents a six-fold coordinated Cr(II) complex containing an external nucleophilic ligand, mostly HCOOMe, that can remain in the Cr coordination sphere also during ethylene polymerization. Another interesting concept proposed in [54] was that Cr(VI) sites might have a role in the first steps of the catalysis in cooperation with the active Cr(II) sites.

Comprehensive studies of the Cr(VI)/SiO_2_ (3 wt% Cr, prepared by thermooxidative treatment at 500 °C) and its activation by ethylene using different methods were conducted by the Wachs and Handzlik groups in 2016 [40]. First, the in situ *k*^2^-weighted, phase-uncorrected Fourier transform (FT) extended X-ray absorption fine structure (EXAFS) experiments provided information about the radial distribution of the atoms surrounding Cr in the starting Cr(VI)/SiO_2_ system: the dehydrated catalyst showed a strong peak at ~1.2 Å from Cr=O and a weak peak at ~1.8 Å from longer Cr–O. The absence of a peak at ~3 Å for Cr–Cr confirmed the presence of isolated Cr(VI)/SiO_2_ sites. The time-resolved in situ Raman spectra of Cr(VI)/SiO_2_ showed strong bands at 986 and 1014 cm^−1^ from the symmetric stretching vibrations of the surface dioxo (≡SiO)_2_CrO_2_ and monooxo (≡SiO)_4_CrO sites; the absence of Raman bands at 200–300 cm^−1^ confirmed the absence of Cr–O–Cr species. During ethylene polymerization, the intensity of the band of (≡SiO)_2_CrO_2_ decreased more rapidly than the band of the (≡SiO)_4_CrO, thus confirming higher reactivity of (≡SiO)_2_CrO_2_ towards ethylene.

Marked results were obtained in the temperature-programmed surface reaction (TPSR) experiment with 1% C_2_H_4_/Ar flow; the temperature was ramped to 800 °C with MS monitoring of the reaction products. During these experiments, the CO_2_ was the only oxidation product detected, with peaks at 280 and 410 °C from (≡SiO)_2_CrO_2_ and (≡SiO)_4_CrO sites, respectively. The estimation of the ratio of dioxo and monooxo species (1.7) showed that relatively high content of the latter cannot be neglected in analysis of the supported Cr(VI)/SiO_2_ catalysts. UV–vis spectral studies during the reaction with ethylene, according to the authors of the work, clearly indicated the formation of Cr(III) sites. During in situ FT-IR spectral studies, a spectral band at 2960 cm^−1^ was detected and assigned to the ν(C–H) of the vinyl reaction intermediate Cr(III)–CH=CH_2_. Further studies of the formation and reactivity of the Cr(III)–CH_2_CH_2_CH=CH_2_ species showed that only part of Cr(III)–CH=CH_2_ species are able to induce ethylene polymerization. Besides fundamentally important spectral studies, fruitful DFT modeling was performed based on proposed (≡SiO)_2_CrOH and (≡SiO)_3_Cr cluster models for ethylene polymerization [40]. In the former case, relatively low activation barriers were found; however, the mechanism of the formation of Cr(III) mononuclear sites remained unclear.

In 2018, Groppo and colleagues studied the activation of Cr(VI)/SiO_2_ by ethylene at 150 °C [55]. They detected an appearance of Cr(III) signals in the EPR spectra during polymerization; however, an increase in the intensity of these signals was in line with the decrease in the intensity of the signals of Cr(V) impurities in the starting Cr(VI)/SiO_2_ sample. It is essential that the Cr(II)/SiO_2_, prepared via reduction of Cr(VI) by CO, was active in ethylene polymerization, but no EPR signs of the presence of Cr(III) were detected. In this way, the Cr(III) nature of the active polymerization sites remained unconfirmed.

**Figure 15:**
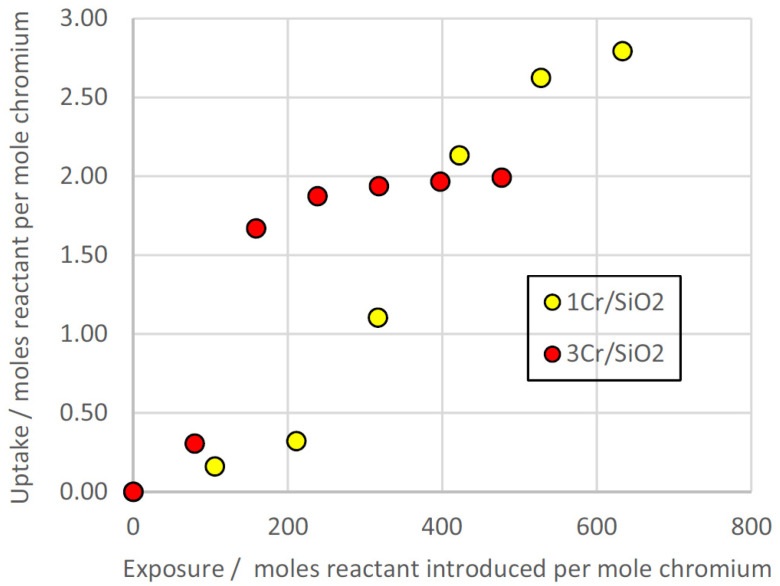
Uptake curves for ethylene during reaction with Cr(VI)/SiO_2_ samples containing 3 and 1 wt% Cr (3Cr/SiO_2_ and 1Cr/SiO_2_, respectively). Ethylene was administered at a temperature of 200 °C in the form of pulses of 1 min duration (40 kPa ethylene diluted in inert gas at atmospheric pressure). Units on both axes are mol/mol. Reprinted with permission from [56]. Copyright (2016) Elsevier B. V.

The study of the reaction of Cr(VI)/SiO_2_ with ethylene at 200 °C [56] showed the formation of oligomers (hex-1-ene, but-1-ene, and internal olefins), with subsequent complex reactions with olefins at elevated temperatures. The stoichiometry of the ethylene uptake was significantly dependent on the Cr content, and saturation at [C_2_H_4_]/[Cr] = 2 was achieved for Cr(VI)/SiO_2_ comprising 3 wt% Cr (Figure 15). It is noteworthy that the temperature-programmed desorption to a temperature of 500 °C led to the formation of the catalyst, more active than the catalyst obtained by standard reduction of the Cr(VI)/SiO_2_ precatalyst with ethylene. A possible explanation for this higher activity might be the formation of “naked” chromium sites after elimination of the ethylene oxidation products, e.g., HCOOMe (see above).

**Figure 16:**
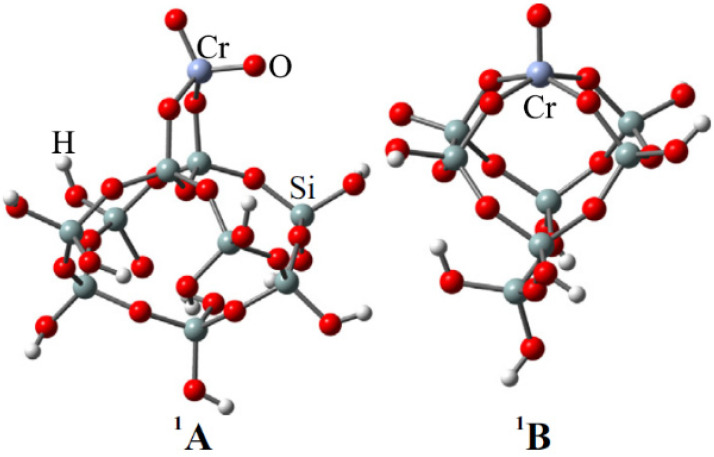
Models of isolated surface Cr(VI) species: **^1^A**—dioxo, **^1^B**—monooxo. Reprinted with permission from [41]. Copyright (2018) Elsevier B. V.

The mechanism of the reduction of Cr(VI)/SiO_2_ by ethylene was the subject of theoretical research of Gierada and Handzlik [41]. Based on the cluster models comprising dioxo **^1^A** and monooxo **^1^B** species (Figure 16), they studied at the PBE0/def2-TZVPP level of DFT a great variety of the reaction mechanisms leading to the formation of different oxidation products.

For dioxo Cr(VI) species, it was found that at the first stage of the reaction of **^1^A** with ethylene, the singlet complex (≡SiO)_2_CrO_2_(π-C_2_H_4_) (**^1^A1**) represents a highly symmetrical structure, whereas in the triplet product **^3^A1** ethylene is bound to the former oxo ligand, and its structure corresponds to the formula (≡SiO)_2_Cr˙(O)OCH_2_CH_2_˙. The energies of **^1^A1** and **^3^A1** turned out to be close, and the activation barriers of the subsequent formation of dioxachromacyclopentane intermediate from **^1^A1** and **^3^A1** were 140 and 63 kJ∙mol^−1^, respectively. Hence, the conventional concerted mechanism of the reductive coupling, proposed by Baker and Carrick [57], is less likely. Further reductive decomposition of the cyclic Cr(IV) intermediate produced two CH_2_O molecules coordinated to Cr(II). Alternative reaction pathways involve ethylene approaches from the Cr–O–Si side with a formation of Cr(VI) oxachromacyclobutane species; the activation barriers amounted to ~160 kJ∙mol^−1^ relative to **^1^A1** for both singlet and triplet species. The further transformations of oxachromacyclobutane may lead to the formation of oxirane or CH_3_CHO. However, the overall ΔG^≠^ for these processes (~160 kJ∙mol^−1^) was higher than the rate-limiting barrier predicted for the reduction to the Cr(II) species with a formation of CH_2_O (127 kJ∙mol^−1^). A hypothetical transformation of oxachromacyclobutane to (≡SiO)_2_Cr(O)=CH_2_ and CH_2_O requires 234 kJ∙mol^−1^ and is hardly feasible. Formaldehyde, formed during the reduction of the Cr(VI) species by ethylene, might undergo subsequent oxidation reactions, but this process seems statistically unlikely. Essentially, more important for the reactivity of PCCs is the transformation of CH_2_O to HCOOMe, observed in [54]. The DFT modeling showed the feasibility of the reaction and, more importantly, the higher stability of the HCOOMe–Cr(II) complex in comparison with the CH_2_O–Cr(II) complex. 

**Figure 17:**
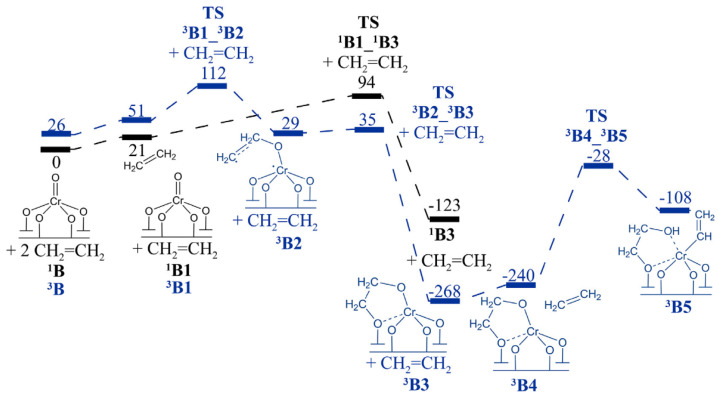
Gibbs energy profile (kJ∙mol^−1^) at T = 373 K for the reduction of the monooxo Cr(VI) species with ethylene and further activation to Cr(IV) vinyl site. Black and blue colors denote singlet and triplet reaction pathways, respectively. Reprinted with permission from [41]. Copyright (2018) Elsevier B. V.

The free energy profile for the reaction of monooxo Cr(VI) species **^1^B** with ethylene is presented in Figure 17. After insertion of the ethylene molecule, very stable Cr(IV) intermediate **^3^B3** formed, which substantiates the lower reactivity of monooxo Cr(VI) sites towards ethylene observed experimentally in previous studies [40].

Recent studies of the photoinduced reduction of Cr(VI)/SiO_2_ by C_2_H_4_ [51] showed that polymerization starts at room temperature with the formation of two different oxidized by-products during the initial steps, HCOOMe and ethylene oxide. While the former was found also during thermally induced reduction, the latter was never observed. The authors proposed that the reason for the oxirane formation might be due to the specific photoactivation mechanism that involves a single oxygen per time passing through the formation of a Cr^V^(=O)(O^−^)* excited state. A second proposed explanation was a lack of oxirane detection due to its desorption at the reaction temperature, but the absence of the oxirane in industrial recyclable feeds [3] does not confirm this hypothesis.

### 4.3. Reduction and Activation by the Olefins Distinct from Ethylene

The use of the nonpolymerizable olefin cyclohexene for the reduction of Cr(VI)/SiO_2_ at room temperature was proposed by Barzan and colleagues in 2016 [58]. The DR UV–vis spectrum of cyclohexene-reduced Cr(VI)/SiO_2_ catalyst (d–d bands at 10,000 cm^−1^ and 16,500 cm^−1^) was interpreted in terms of Cr(II) sites in a six-fold coordination. The Cr K-edge XANES spectrum did not allow for definitive causal conclusions about the nature of the Cr species formed; EPR studies indicated that the amount of Cr(III) species is below 0.5% of the total chromium sites. Based on these data, the products of the reduction by cyclohexene were identified as Cr(II) complexes, coordinated with additional ligands, the products of the oxidation of cyclohexene. Unfortunately, these products were not separated and analyzed, even when trying to hydrolyze the samples [58]. It was significant that cyclohexene-reduced Cr(VI)/SiO_2_ catalyzed ethylene polymerization without an induction period and with no change in the DR UV–vis and XANES spectra. Despite the high sensitivity of the DR UV–vis and XANES techniques, the possibility that a fraction of active chromium sites is below the sensitivity of both methods cannot be definitely discarded, and it might even be argued that the active sites are the few Cr(III) species detected by EPR only. On the other hand, one cannot fail to assume that Cr(II) species, formed during the reduction of Cr(VI)/SiO_2_ by cyclohexene, are already in “good” electronic and geometric configuration to efficiently polymerize ethylene.

In the same year, the results of the study of the reduction of Cr(VI)/SiO_2_ by olefins at elevated temperatures were published by McDaniel and colleagues [56]. When using hex-1-ene or cyclohexene at 150 °C, the reactions proceeded toward a monolayer coverage with near 1:1 stoichiometry, and low-active catalysts were obtained. In a continuation of the research, McDaniel’s group studied the reduction of synthesized Cr(VI)/SiO_2_ (0.1, 1.0, and 3.0 wt% Cr) and commercial Cr(VI)/Ti/SiO_2_ (1.0 wt% Cr and 2.5 wt% Ti) catalysts by different olefins. Oxidation of ethylene and α-olefins resulted in the formation of esters, carboxylic acids, aldehydes, and ketones (including the products of C=C bond cleavage); pent-2-ene also formed the products of C=C bond cleavage, but oxidation of cyclohexene resulted in the formation of cyclic products, while no carboxylates formed.

To verify the traditional view on active site formation that involves the displacement of the aldehydes from Cr(II) by ethylene, a number of experiments were conducted with Cr(II)/SiO_2_, and it was demonstrated that hexan-2-one, hexanal, and alcohols, being added at equimolar ratio, deactivated the catalyst completely [56]. However, the addition of 1 eq. AcOH resulted in the formation of a catalyst that was very close to ethylene-reduced Cr(VI)/SiO_2_ in activity. It was proposed that carboxylates, formed during the reduction of Cr(VI)/SiO_2_, have to be considered as permanent ligands under typical polymerization conditions.

### 4.4. Reduction and Activation by Nonolefinic Hydrocarbons

**Figure 18:**
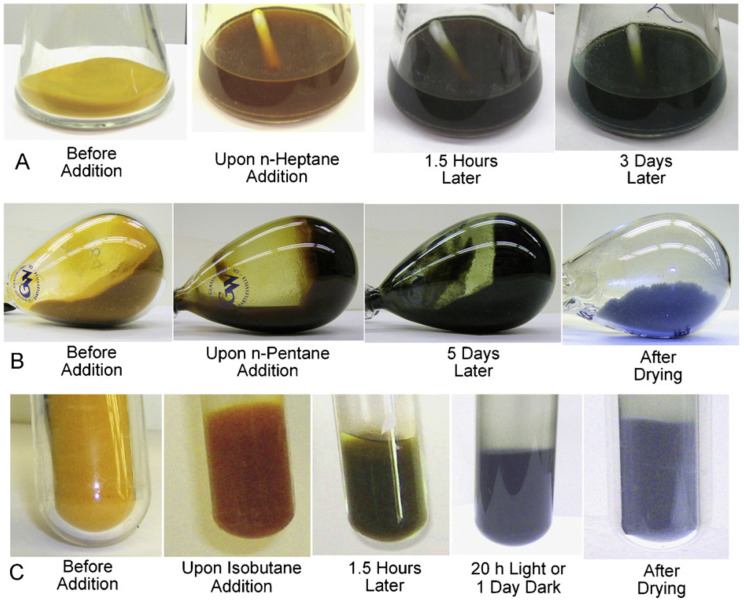
Cr(VI)/silica–titania catalysts reduced at 25 °C in the presence of (**A**) *n*-heptane, (**B**) *n*-pentane, and (**C**) isobutane. Reprinted with permission from [59]. Copyright (2012) Elsevier B. V.

In 2012, McDaniel and colleagues reported somewhat unexpected results of the study of the interaction of Cr(VI)/SiO_2_ with *n*-heptane, *n*-pentane, or isobutane [59]. Surprisingly, even at 25 °C, these hydrocarbons oxidized with a formation of the corresponding ketones (and traces of secondary alcohols) or *tert*-butanol, with clearly visible changes in the color of the catalyst (Figure 18). Exposition by alkane vapors at 90–105 °C resulted in the formation of active catalysts. However, these catalysts were inferior to the benchmark in terms of productivity and initiated the formation of PEs with lower MW. This finding is of great importance for manipulations with PCCs in the industry with the use of aliphatic hydrocarbons as diluents or reaction media. Also note that the reduction of PCCs by hydrocarbons was found to be accelerated by UV light. As a result, McDaniel and colleagues proposed the alternative use of PCCs as recoverable agents for the oxidation of hydrocarbons [60,61].

Follow-up studies of McDaniel’ group [62] are of fundamental importance for the understanding of the mechanisms of PCC activation. They noticed the absence of the CH_2_O or other oxygenated by-products in the recycle streams of industrial reactors. During laboratory studies, the elimination and detection of oxygenates from olefin-reduced Cr(VI)/silica required hydrolysis of the spent catalyst. When studying the activation of Cr(VI)/SiO_2_ by saturated hydrocarbons, McDaniel and colleagues showed experimentally that the products of the oxidation of saturated hydrocarbons and toluene by the Cr(VI) form of a TiO_2_-modified PCC represent complex mixtures of the products (Table 2).

**Figure 19:**
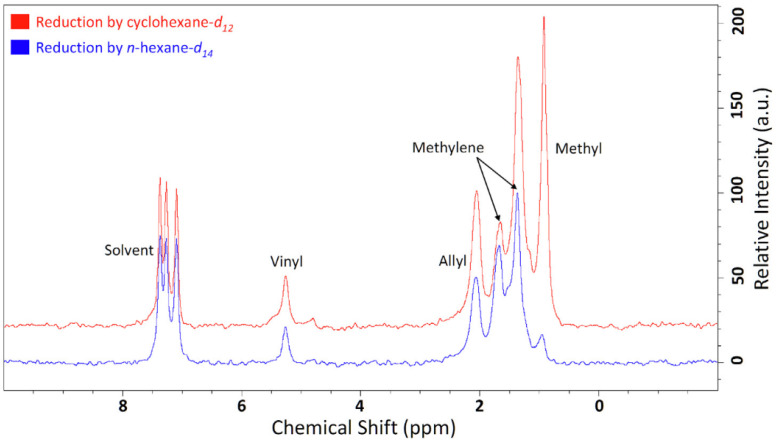
^2^H NMR spectra of PE made by two hydrocarbon-reduced catalysts. Reprinted with permission from [62]. Copyright (2021) Elsevier B. V.

Furthermore, and more important for the understanding of the processes of the activation of PCCs in the industry, the fragments of saturated hydrocarbons were found to become initial chain fragments of the PE formed (Figure 19).

**Scheme 4:**
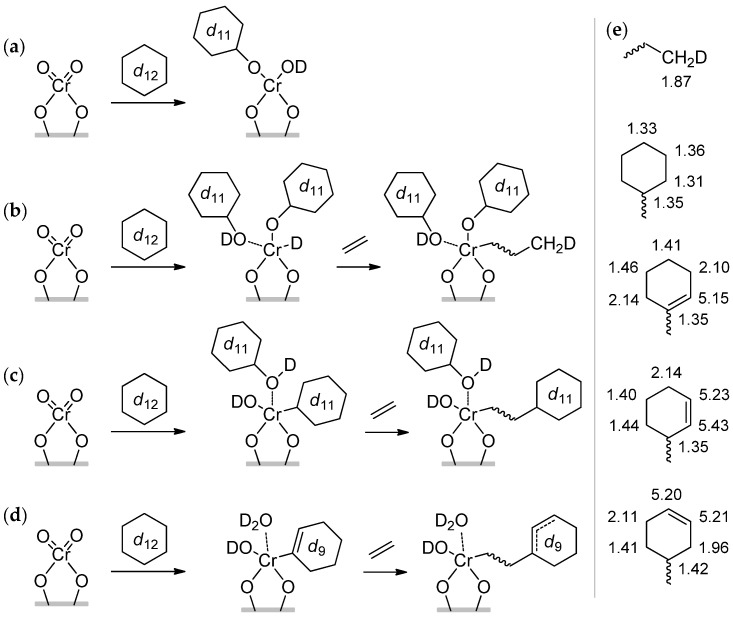
(**a**–**d**) Possible pathways of the reduction and activation of Cr(VI)/SiO_2_ using cyclohexane-*d*_12_; (**e**) chemical shifts of the ^2^H-labeled end groups [62].

As can be seen in Scheme 4, cyclohexane-*d*_12_ could possibly be incorporated into the polymer chain by three out of four possible reaction pathways. In each pathway, the Cr(VI) reacts with a C–D bond on the cyclohexane-*d*_12_. In the upper pathway (a), the cyclohexane-*d*_12_ does not alkylate the Cr. In the pathways (b), (c), and (d), Cr(VI) is reduced by cyclohexane-*d*_12_ to yield three different deuterated products, each of which can then initiate the growth of the PE chain after the addition of CH_2_=CH_2_. In pathway (b), C–D bond cleavage occurs with a formation of Cr–D species that are able to coordinate and insert an ethylene molecule with the formation of a –CH_2_D end group containing PE. Similarly, in pathway (c), the Cr-alkyl group is the cyclohexyl group, which can potentially also begin the first chain upon ethylene polymerization. Finally, pathway (d) is similar to (c) except that the initiating group is a cyclohexenyl fragment. The pathways (c)–(d) could potentially result in five different initiating D species on the first polymer chain (Scheme 4e). The ^2^H NMR shifts expected from each deuterium position, calculated using Mnova Suite Chemist (Mestrelab Research, Santiago de Compostela, Spain), are also shown in Scheme 4. As can be seen from Figure 19 and Scheme 4e, the reaction pathways (b)–(d) are realized after activation of Cr(VI)/SiO_2_ by cyclohexane-*d*_12_. Experiments with *n*-hexane-*d*_14_ resulted in similar observations. 

In this way, in their study, McDaniel and colleagues showed that the importance of the factor of reaction media should not be underestimated with regard to industrial processes. Cr(VI)/SiO_2_ can be reduced by saturated hydrocarbons with a formation of active Cr–alkyl species containing different oxygenated ligands, which further increases the diversity of the catalytic centers.

**Figure 20:**
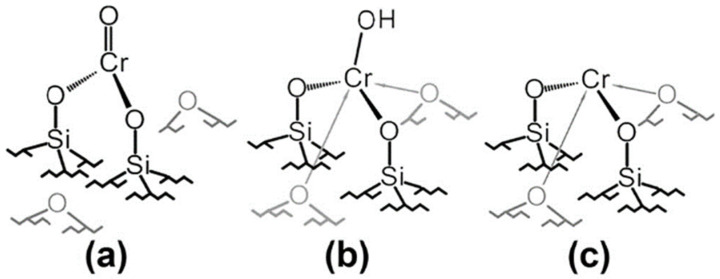
Pictorial representation of the local structures for the reduced Cr species on CH_4_-Cr/SiO_2_ catalyst, as suggested by spectroscopic methods. (**a**) Cr(IV) sites, (**b**) Cr(III) sites, (**c**) Cr(II) sites. Reprinted with permission from [63]. Copyright (2019) Elsevier B. V.

In 2019, McDaniel and colleagues investigated a prereduction of Cr(VI)/SiO_2_ by CH_4_ at 400 °C [63]. According to industrial reports [3,7], this procedure resulted in elimination of the induction period and ~30% increase in the catalytic activity, without substantial changes in PE characteristics. The release of HCOOH and H_2_ was detected during prereduction. Based on *operando* Cr K-edge XANES and in situ DR UV–vis-NIR data, the Cr site distribution was found as follows: Cr(IV) (active, 44%), six-fold Cr(III) (inactive, 33%), and Cr(II) (active, 23%) (a, b, and c, respectively, in Figure 20).

### 4.5. Organoaluminum Activators

With regard to PCCs, aluminum alkyl activators can act as a scavenger to remove impurities in the polymerization system and redox by-products attached to Cr species, reducing reagents for Cr(VI) with a formation of lower valent species and alkylating agents for reduced Cr species; the use of R_3_Al activators with PCCs is well-known and is applied in industry [3,4,5,8], but their interaction with Cr sites at the molecular level is not completely understood.

In recent years, relatively few studies have been carried out using organoaluminum activators. In particular, in 2017, Terano and colleagues presented the results of a comparative study of Et_3_Al, ^i^Bu_3_Al, (*n*-C_8_H_17_)_3_Al, and (*n*-C_8_H_17_)_2_Al(OAr) (where Ar = 2,6-di-*tert*-butyl-4-methylphenyl) [64], which revealed the preference of (*n*-C_8_H_17_)_3_Al in terms of increasing the activity and suppressing the methyl branch formation.

**Figure 21:**
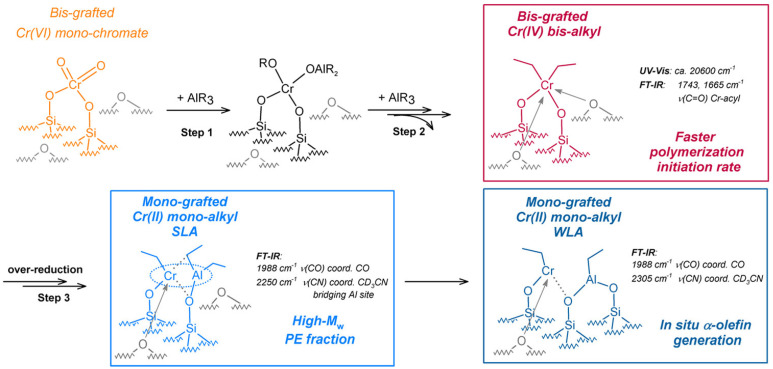
Representation of the structure at a molecular level of the Cr species detected by spectroscopic methods on Cr(VI)/SiO_2_ + Et_3_Al (1:2). Reprinted with permission from [65]. Copyright (2020) American Chemical Society.

In 2020, Groppo and colleagues reported the results of the systematic study of the effect of Et_3_Al on the Cr(VI)/SiO_2_ catalyst [65]. They found that at an Al/Cr ratio of 2:1, only ~50% of the starting Cr(VI) sites are reduced, with the formation of (1) Cr(IV) bis-alkyl sites, which are the major actors in the polymerization of ethylene, explaining the faster polymerization initiation rate; (2) six-fold coordinated Cr(III) sites, whose amount does not correlate with the catalyst activity; and (3) two types of monografted Cr(II) sites, having weak and strong Lewis acid characters, respectively. The former Cr(II) sites are responsible for in situ α-olefin generation (and hence SCB formation) and the latter Cr(II) sites account for the formation of the high-MW polymer fractions (Figure 21). The nature of these sites was confirmed by DR UV–vis–near-IR, EPR, and FT-IR spectroscopy methods using CO and MeCN probes. The dominant role of Cr(IV) bisalkylated sites in ethylene polymerization allows researchers to suggest that similar species might actually be the active sites in ethylene polymerization on the unmodified Cr(II)/SiO_2_. Note that a self-alkylation mechanism involving the formation of Cr(IV) bisalkyl sites starting from Cr(II) was also supported by the studies of McDaniel and colleagues [66] (see below).

It is noteworthy that in 2020, Weckhuysen and colleagues published the results of similar research of Cr(VI)/SiO_2_ activation by Et_3_B and Et_3_Al [67]. Besides trivial catalytic results (reduction of the induction period, higher catalytic productivity at initial polymerization stages), the results of the study of the Cr sites reduced by 1.5 and 10 equivalents of Et_3_M were presented. The main function of Et_3_B (1.5 eq.) was a reduction of Cr(VI)/SiO_2_ to Cr(II)/SiO_2_, whereas in the case of Et_3_Al (1.5 eq.), different Cr sites were detected, in good agreement with the results of Torino’s group [64]. Weckhuysen noted that Et_3_Al, in contrast with R_3_B, acted not only as a reductant but also as a reducibility enhancer in the reaction of Cr(VI)/SiO_2_ with ethylene. The addition of Et_3_B and Et_3_Al affected the properties of PE obtained using PCCs [68], but this aspect is not a subject of our review.

In summary, complete clarity has been achieved only on the issue of the activation of Cr(VI)/SiO_2_ by CO, which results in the formation of well-defined Cr(II)/SiO_2_ species. Activation of Cr(VI)/SiO_2_ by ethylene may lead to the formation of Cr(III)–alkyl (or Cr(III)–vinyl), but the results of the recent experimental works clearly point to the possibility of the formation of Cr(II) and Cr(IV) active sites during activation of Cr(VI)/SiO_2_ by ethylene, olefins, saturated hydrocarbons, and trialkylaluminum.

## 5. Activation of the Cr(II)/SiO_2_

In this section, we limit ourselves to the description and analysis of the studies based on an assumption that the active species, formed after activation/reduction of the Cr(VI)/SiO_2_ by CO (or ethylene—in accordance with the debated opinions of the authors of the corresponding works), represent Cr(II) complexes. At present, it becomes clear that the reduction of Cr(VI)/SiO_2_ by ethylene is unlikely to result in “naked” (≡SiO)_2_Cr(II) sites, and a correct comparison of experimental and theoretical studies is possible only for CO-reduced Cr(VI)/SiO_2_ systems. 

### 5.1. Activation by Ethylene

**Scheme 5:**
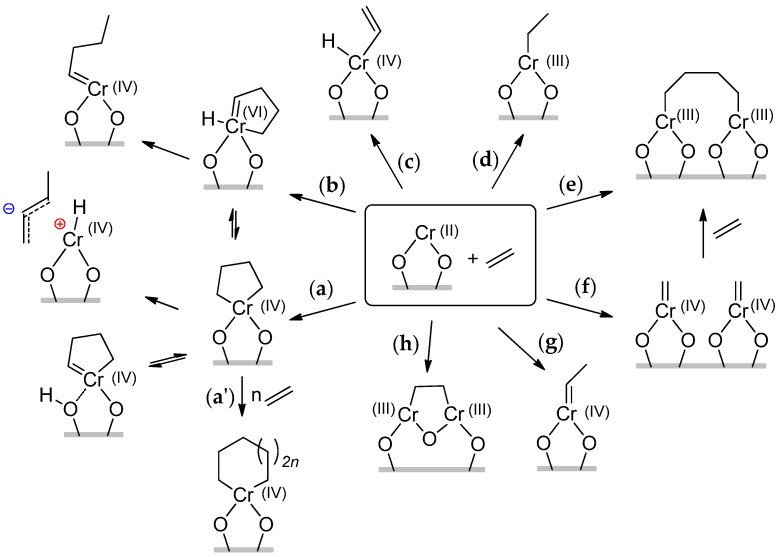
Various proposed initiation mechanisms for ethylene polymerization over Cr(II)/SiO_2_.

As applied to PCCs, by “activation”, we mean the formation of active catalytic centers that are able to maintain the growth of the PE chain. It is the activation stage that determines the nature of the catalytic center capable of the further coordination and insertion of the ethylene molecule, i.e., the true polymerization mechanism. And, that is, the activation stage is not completely understood to date. In many previous works and reviews, various hypotheses have been proposed to explain the nature of the catalytic centers in PCCs and the chemistry of their formation. The possible mechanisms of the formation of active species, which were clearly generalized in [8], are presented in Scheme 5 with the relevant commentary (see below).

In early experiments on the interaction of CO-reduced Cr(VI)/SiO_2_ with deuterated ethylene, Ruddick and Badyal detected the formation of fully deuterated hex-1-ene at the initial stage of the process, thereby proving that no additional hydrogen atoms (e.g., from the surface silanols) are required at the initiation step [69]. This finding was viewed by them as an argument in favor of metallacycle formation and subsequent chain propagation by a Green–Rooney-type mechanism (Scheme 5, pathways a, b). To date, the active sites concerned with Cr-alkylidene species (Scheme 5, pathways a, b, f, g) still lack conclusive evidence. The most obvious “pure” metallacyclic mechanism of chain propagation (Scheme 5, pathway a with subsequent coordinations and insertions of ethylene molecules a’) has not been confirmed in recent studies. The models involving Cossee–Arlman chain propagation (Scheme 5, pathways c, d) still hold the most popularity; however, the formation of Cr(III) alkyl species (Scheme 5, pathway d) requires the involvement of the additional H atom from an unknown source (the problem of the “extra” hydrogen atom mentioned above).

Studies of the mechanisms of the activation of PCCs (especially early laboratory investigations) were often conducted under conditions that are far away from actual industrial conditions, and this aspect should be taken into account when comparing the results of research. In this section, we discuss the results of recent experimental and theoretical studies of the activation of PCCs as an attempt to clarify the nature of the catalytic species that are able to polymerize ethylene with high rates.

It is appropriate to mention here the results of the study of Groppo et al. [70], which showed that at low temperatures and ethylene loadings comparable to the number of Cr(II) sites, two “anomalous” bands at 2931 and 2861 cm^−1^ in FT-IR spectra were clearly detected and attributed to the formation of small metallacycles (Scheme 5, pathway a → a’). The formation of similar species could not be excluded at early polymerization stages, but the metallacycle mechanism seems hardly applicable for the further polymerization of ethylene on PCCs (see below).

**Figure 22:**
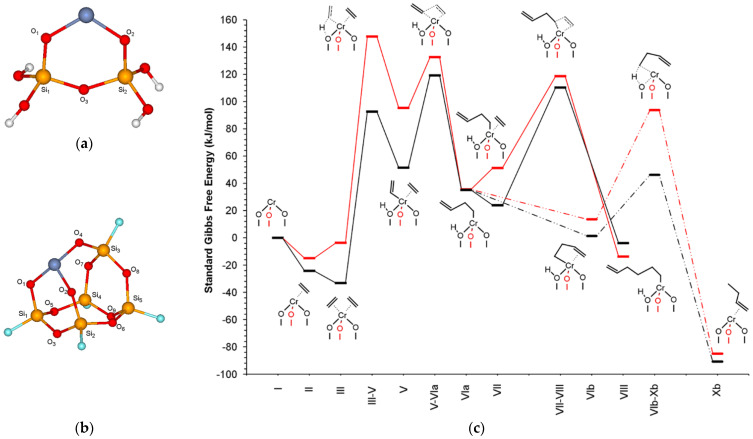
Structure of the optimized (**a**) Cr(II) cluster and (**b**) Cr(III) cluster; (**c**) comparison of proton-transfer-initiated alkylation (solid lines), subsequent Cossee–Arlman insertion (solid lines), and chain termination (dashed lines) for Cr(II) (black) and Cr(III) (red). Reprinted with permission from [71]. Copyright (2015) American Chemical Society.

In 2015, Fong et al. carried out DFT modeling (ωB97X-D) of different mechanisms of activation of Cr(II) and Cr(III) sites using simplified models (Figure 22a,b) [71]. They found that previously proposed chromacycle ring expansion and Green–Rooney mechanisms should be ruled out due to prohibitively high activation barriers for propagation. On sites with adjacent bridging hydroxyls, either ≡Si(OH)Cr^II^–alkyl or ≡Si(OH)Cr^III^–alkyl, initiated by proton transfer from ethylene, chain growth by a Cossee–Arlman-type mechanism was found to be fast (Figure 22c). However, the initiation step was uphill and extremely slow, and termination was faster than propagation (due to the formation of the oligomerization sites). A monoalkylchromium(III) site without an adjacent proton, (≡SiO)_2_Cr–alkyl, was found to be viable as an active site for polymerization (for (≡SiO)_2_Cr–*^n^*Bu, calculated relative free energies were 73.5 and 80.4 kJ∙mol^−1^ for ethylene coordination and insertion, 81.0 kJ∙mol^−1^ for β-hydride elimination, and 106.2 kJ∙mol^−1^ for β-hydride transfer to ethylene), although its precise origin and formation route remained unknown.

As was shown by Brown et al. in 2015, the Cr(II) sites react quantitatively with ethylene at 80 °C to generate the (organo)Cr(III) sites, as evidenced by the concomitant appearance of resonances associated with Cr(III) in the Kramers region of the EPR spectrum, X-ray absorption, and UV–vis spectroscopy data [47]. After “titration” by ethylene, the data of elemental analysis showed the [C]/[Cr] ratio of ~2:1, thus suggesting that at the end point of the titration, no further changes were observed in the UV–vis spectrum; the reaction product has taken up one ethylene per Cr site. These sites reacted quantitatively with the generation of (organo)Cr(III) sites that can polymerize ethylene, suggesting that the Cr(III) species are the initiating sites.

**Figure 23:**
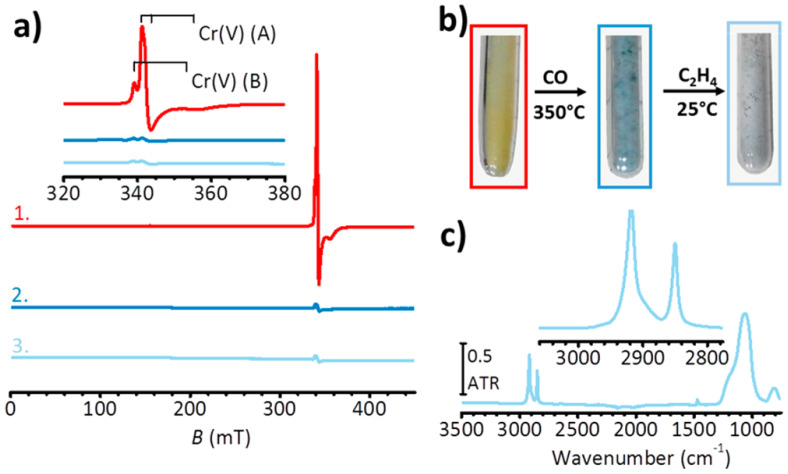
(**a**) X-band CW-EPR spectra of Cr(VI)/SiO_2_ (1), CO-reduced Cr(II)/SiO_2_ (2), and Cr(II)/SiO_2_ after reaction with ethylene (P = 100 mbar) at room temperature (3). The enlargement of the g = 2 resonant-field region is shown in the inset. The spectra were recorded at T = 77 K, with microwave power of 1 mW. (**b**) Pictures of the Cr/SiO_2_ catalyst upon the different treatments. The colors correspond to those of the EPR spectra in panel (**a**). (**c**) ATR FT-IR spectrum of the Cr(II)/SiO_2_ catalyst after ethylene polymerization. The inset shows a magnification of the ν_asym_(CH_2_) and ν_sym_(CH_2_) bands of polyethylene. Reprinted with permission from [55]. Copyright (2018) American Chemical Society.

These results were notably corrected in more recent work of Groppo and colleagues [55]. They showed that Cr(II)/SiO_2_, prepared by the reduction of Cr(VI)/SiO_2_ (containing ~2% of Cr(V)) with CO, polymerizes ethylene at 25 °C without the formation of Cr(III) species; no signals were detected in EPR spectra during polymerization (Figure 23).

Although the absence of an EPR signal does not necessarily imply that no Cr(III) is present in the sample, considering the EPR detection limit, this should be much below 10 ppm (<0.2% of the total Cr sites). It seems, therefore, unrealistic that such a small amount of Cr(III), if present, is only responsible for the catalyst activity.

**Scheme 6:**
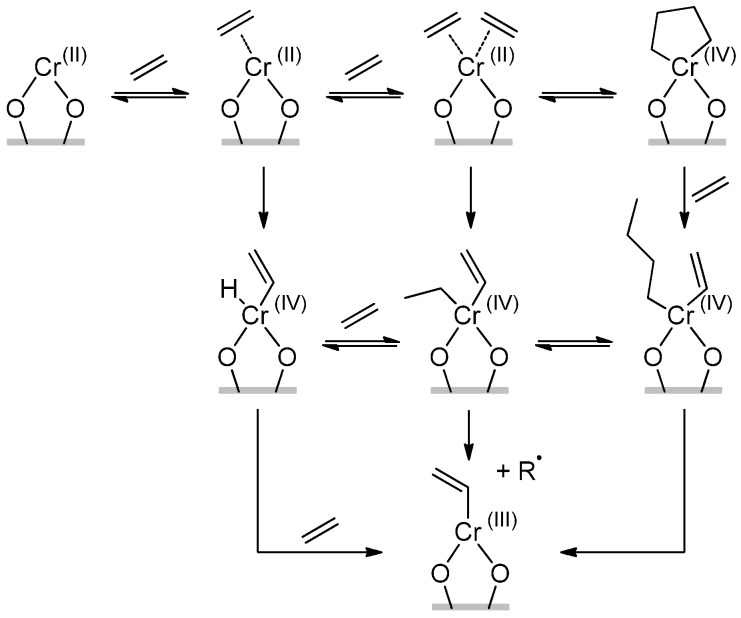
Mechanisms investigated for ethylene activation in two silica-supported Cr(II) sites (II and III) to form various (organo)Cr(IV) sites, which react to yield an alkyl radical R^∙^ and the active site RCr(III) [72].

In a later article of Scott and colleagues [72], the mechanism of the activation of Cr(II)/SiO_2_ with a formation of Cr(III) species and free alkyl radicals was assessed in comparison with other possible mechanisms (Scheme 6). An idea about the formation of free radical intermediates during Cr(II) activation was proposed by Kissin and Brandolini back in 2008 [73].

**Figure 24:**
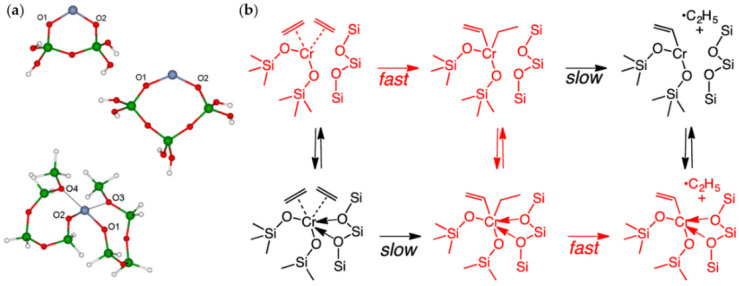
(**a**) Model Cr(II) clusters; (**b**) concerted H atom transfer followed by Cr–C bond homolysis in Cr/SiO_2_ sites with hemilabile siloxane ligands. H atom transfer is much faster when the siloxanes dissociate, but the homolysis rate is strongly promoted by their interaction with Cr. Structures in red show a possible path for fast site activation involving reversible “on-demand” coordination of hemilabile siloxane ligands. Reprinted with permission from [72]. Copyright (2016) American Chemical Society.

Three model Cr(II) clusters were used for calculations (Figure 24a); they represented “small” and “large” chromasiloxane rings and also the cluster containing four-coordinated Cr(II). DFT modeling for every structure separately did not allow the creation of energy profiles with reasonable activation barriers. However, a combination of the modeling for two- and four-coordinated Cr(II) species allowed researchers to propose the mechanistic concept presented in Figure 24b. The main drawback of this (and similar) model is a high activation barrier for oxidative insertion of two ethylene molecules with a formation of (≡SiO)_2_Cr(CH=CH_2_)Et species (~150 kJ∙mol^−1^).

**Figure 25:**
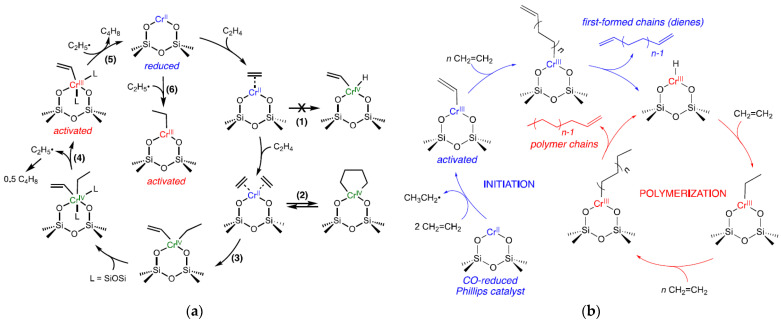
(**a**) Possible mechanisms for the formation of the vinyl-Cr(III) initiating site in the Phillips catalyst; (**b**) proposed mechanism for initiation followed by ethylene polymerization over the Phillips catalyst. Reprinted with permission from [74]. Copyright (2017) American Chemical Society.

The study of Scott and colleagues [74] uncovered yet more evidence of the formation of Cr(III) species during the reaction of (≡SiO)_2_Cr with ethylene. During investigations, an optically transparent monolith of Cr(VI)/SiO_2_ was reduced by CO to the spectroscopically determined Cr(II)/SiO_2_. Ethylene caused rapid reoxidation of Cr(II) sites to Cr(III) sites (as evidenced by EPR studies), containing Cr–CH=CH_2_ fragments according to solid-state ^13^C CP-MAS NMR (two signals at 75 and 146 ppm), FT-IR, and Raman spectral data. These species were found to be capable of initiating ethylene polymerization even at subzero temperatures. The proposed mechanism (Figure 25a) implies the one-electron oxidation of Cr(II) via ethylene disproportionation. The accompanying formation of organic radical intermediates was confirmed by EPR and by GC analysis of the reaction products with the detection of *n*-butane as a main component. The authors proposed that the further ethylene coordinations/insertions result in PE formation, but the first-formed PEs should be α,ω-dienes (Figure 25b). The proposed reaction scheme looks appealing, but a too-low experimental temperature of ethylene activation is so poorly correlated with the calculated activation barrier (~150 kJ∙mol^−1^) [72]. In addition, the formation of α,ω-dienes can and have to be proved experimentally. This was not performed.

**Figure 26:**
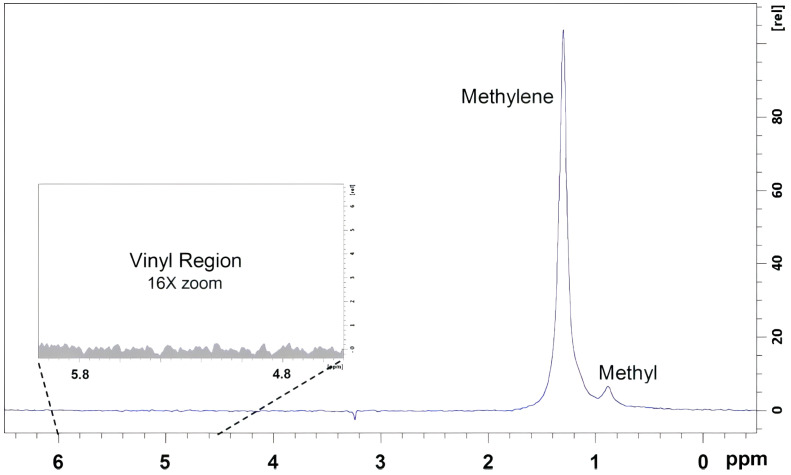
Deuterium NMR spectrum of the labeled polymer, from preadsorption of C_2_D_4_ onto Cr(II) catalyst, followed by polymerization of C_2_H_4_. Reprinted with permission from [66]. Copyright (2019) American Chemical Society.

The mechanistic concept, proposed in [72,74], is attractive due to the fact that it allows the solving of the problem of “extra” hydrogen during the formation of the first PE chain. In 2019 [66], McDaniel and colleagues reported the results of experimental verification of the mechanism, presented in Figure 25b. To detect the very fact of initiation of ethylene polymerization by Cr–vinyl species, they preadsorbed *isotopically labeled* ethylene or propylene onto a Cr(II)/Ti/SiO_2_ catalyst at −78 °C and conducted a series of polymerization experiments with C_2_H_4_ at 0 and 80 °C. ^2^H NMR (Figure 26) studies of the PE, synthesized using a C_2_D_4_–Cr(II)/Ti/SiO_2_ initiator, clearly showed the absence of ^2^H-labeled vinyl end groups; ^1^H and ^13^C NMR spectral data confirmed this conclusion.

**Scheme 7:**
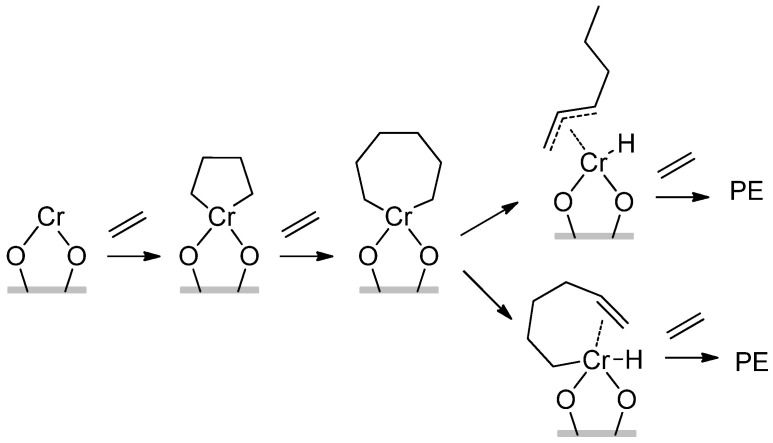
Possible formation of an allyl or ω-alkenyl ligand that donates the starting hydrogen.

To explain these experimental results, McDaniel and colleagues proposed the existence of a prior reaction between a monomer and Cr to donate the extra hydrogen. This “H-donor compound” could be a ligand that stays on the Cr site during polymerization, such as allyl or ω-alkenyl, as illustrated in Scheme 7. Both reactions leave a Cr–H (or Cr–D) starting group for the first chain (or an ethyl group, if the H transfer to incoming ethylene occurs). But in the grand scheme of things, the detailed mechanism of the initiation of polymerization of ethylene by Cr(II)/SiO_2_ remains unclear even after this remarkable research.

**Scheme 8:**
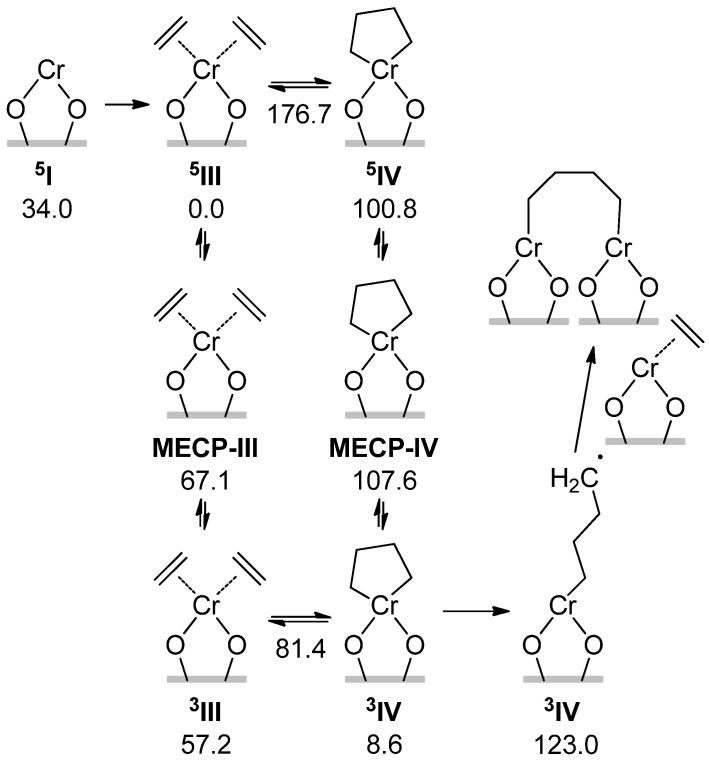
Creation of a pair of Cr(III) active sites from Cr(II) species via intermediate metallacycle formation [75]. The free energies relative to **^5^III** are given in kJ∙mol^−1^.

The metallacyclic mechanism, analyzed earlier by Scott, Peters, and colleagues using the simplest chromadisiloxane model [71,72], once again aroused the attention of this scientific group in 2018 [75]. They proposed that the metallacycle mechanism can be further extended by the participation of free radical intermediates. The results of the calculations (ωB97X-D/def2-TZVP and TZVP level of theory) are presented in Scheme 8. The most stable form **^5^III** of the (≡SiO)_2_Cr(π-C_2_H_4_)_2_ complex has quintet spin multiplicity; its direct conversion to the quintet metallacycle **^5^IV** required overcoming a free energy barrier of 177 kJ∙mol^−1^, while a spin-crossing route to the triplet metallacycle **^3^IV** required only 81 kJ∙mol^−1^. The chromacyclopentane site IV can undergo direct homolysis of the Cr–C bond with a formation of a tethered *n*-butyl radical **V**. The latter, if **^5^V** is sufficiently close to another Cr(II) with an open coordination site, could react with that site to form a 1,4-butylene bridge. The overall computed barrier for this initiation process is 132 kJ∙mol^−1^, and Poisson statistics suggest that this mechanism could activate ∼35% of Cr sites on a commercial catalyst with a loading of 0.4 Cr∙nm^−2^.

**Scheme 9:**
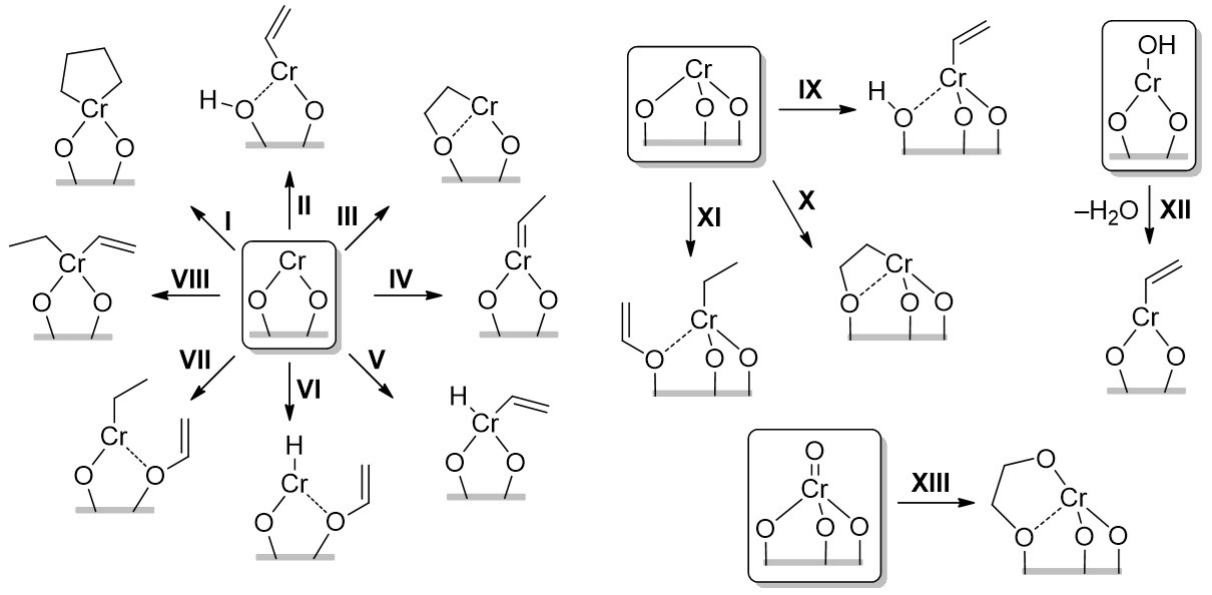
Potential pathways for the active site formation from Cr(II), Cr(III), Cr(III)–OH, and Cr(V) oxide precursors upon contact with ethylene, considered in [76].

**Figure 27:**
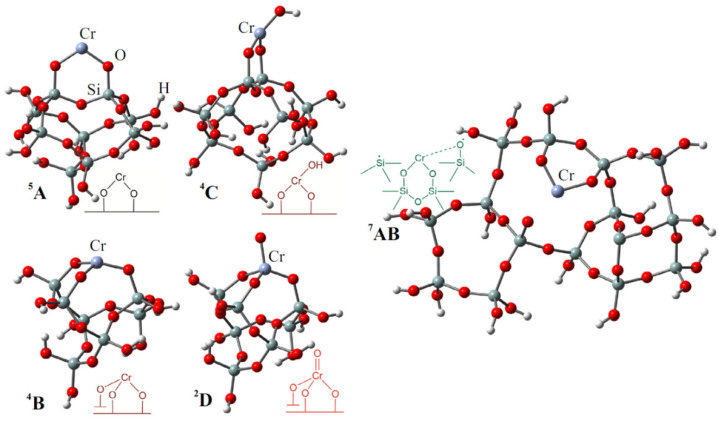
Optimized models of isolated surface Cr sites: **^5^A**—Cr(II), **^4^B**—Cr(III), **^4^C**—Cr(III)–OH, **^2^D**—Cr(V), and **^7^AB**—Cr(II) with adjacent surface defects. The superscript numeral before the letter shows the spin state. Reprinted with permission from [76]. Copyright (2017) Elsevier B. V.

Mechanistic insights of the formation of active Cr sites were the subject of further studies of Gierada and Handzlik. In [76], they analyzed at PBE0/def2-SVP level of theory 13 possible activation pathways from (≡SiO)_2_Cr, (≡SiO)_3_Cr, (≡SiO)_2_CrOH, and (≡SiO)_2_Cr=O species (Scheme 9) with the use of cluster models (Figure 27).

**Figure 28:**
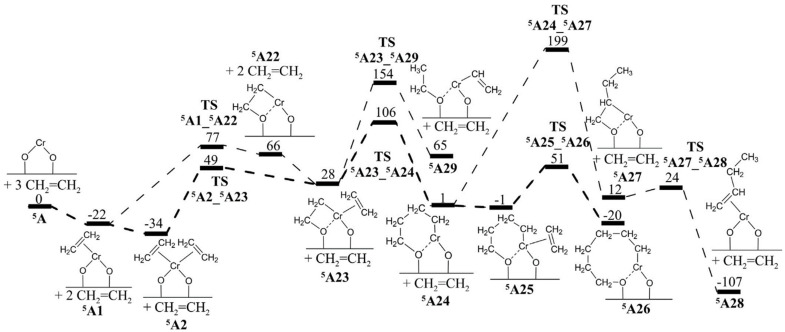
Gibbs energy profile (kJ∙mol^−1^) at T = 373 K for the oxachromacycle ring expansion mechanism over Cr(II) oxide species (quintet reaction pathway). The thicker line represents the preferred route. Reprinted with permission from [76]. Copyright (2017) Elsevier B. V.

Ethylene transformations over Cr(II) species: Ethylene coordination to the species **^5^A** was found to be thermodynamically preferred (ΔG = −22 and −12 kJ∙mol^−1^ for binding with the first and the second C_2_H_4_ molecules). For (≡SiO)_2_Cr(π-C_2_H_4_) adduct **^5^A1**, the proton transfer from π-bonded ethylene (Scheme 9, pathway **VI**) turned out to be endergonic (178 kJ∙mol^−1^) with an extremely high activation barrier (301 kJ∙mol^−1^); the intermediates (≡SiO)_2_Cr(H)(CH=CH_2_) and (≡SiO)_2_Cr=CHMe (Scheme 9, pathways **V** and **IV**) were also unlikely (ΔG = 214 and 248 kJ∙mol^−1^ relative to **^5^A1**, respectively). A much lower activation barrier (143 kJ∙mol^−1^) was predicted when proton transfer from ethylene generates (≡SiO)(≡SiOH)CrCH=CH_2_ (Scheme 9, pathway **II**). Further transformations of this intermediate (coordination and insertion of the second C_2_H_4_ molecule) resulted in formation of a but-1-ene π-complex with **^5^A** via low-energy TS (27 kJ∙mol^−1^); the chain propagation required over three times higher ΔG^≠^ = 91 kJ∙mol^−1^. In this way, the mechanism involving (≡SiO)(≡SiOH)CrCH=CH_2_ might explain the formation of low-MW oligomers, mainly but-1-ene. The chromacycle formation (Scheme 9, pathway **I**) required 83 kJ∙mol^−1^, and further ring expansion was found to be exergonic (−69 kJ∙mol^−1^.) with ΔG^≠^ = 129 kJ∙mol^−1^. However, hex-1-ene elimination from chromacycloheptane was found to be preferable in comparison with the further coordination/insertion of a C_2_H_4_ molecule. The formation of oxachromacycle (Scheme 9, pathway **III**) was endergonic (66 kJ∙mol^−1^) with ΔG^≠^ = 99 kJ∙mol^−1^, and lower barriers and energies were obtained for the (≡SiO)_2_Cr(π-C_2_H_4_)_2_ adduct (Figure 28). Further ethylene coordinations/insertions required overcoming relatively low barriers without spin crossing (in contrast with the chromacycle mechanism). In this way, the oxachromacycle ring expansion mechanism (Figure 28) cannot be excluded as the potential route for ethylene polymerization, taking into account that the calculated overall activation barrier for the initial stage is not very high (140 kJ∙mol^−1^), and the following propagation stages are much faster and clearly kinetically preferred over the termination stages.

High activation barriers were obtained for the transformation of (≡SiO)_2_Cr(π-C_2_H_4_)_2_ to CrEt and Cr(CH=CH_2_)Et species (Scheme 9, pathways **VII** and **VIII**); for the latter species, ΔG^≠^ for coordination/insertion of C_2_H_4_ was estimated by the value of 150 kJ∙mol^−1^, which is higher than the barriers determined for other initiation mechanisms starting from **^5^A1**. In this way, among Cr(II)-based routes, only oxachromacycle route **III** appears feasible.

Cr(III) oxide species three-fold bonded to the surface (model **^4^B**, Figure 27), a potential active site precursor for ethylene polymerization [71,77,78], were also examined in [76]. Similar Cr(III) sites on silica can be easily and irreversibly formed from Cr(II) species and neighboring surface defects. Coordination of the first C_2_H_4_ molecule to the species **^4^B** was a less exergonic process (ΔG = −4 kJ∙mol^−1^) than in the case **^5^A** (ΔG = −22 kJ∙mol^−1^); coordination of the second C_2_H_4_ molecule was endergonic (ΔG = 26 kJ∙mol^−1^). The calculated overall activation barrier for the formation of Cr(III)–CH=CH_2_ species (Scheme 9, pathway **IX**) was 161 kJ∙mol^−1^, being higher than the activation barriers obtained for some other initiation mechanisms. Very high ΔG^≠^ = 247 kJ∙mol^−1^ was obtained for the formation of Cr(III)-Et with the surface ligand ≡SiOCH=CH_2_ (Scheme 9, pathway **X**). The oxachromacycle mechanism (Scheme 9, pathway **X**), similar to the one presented in Figure 28, has a right to exist, but the overall activation Gibbs energy was found to be much higher (149 kJ∙mol^−1^).

**Figure 29:**
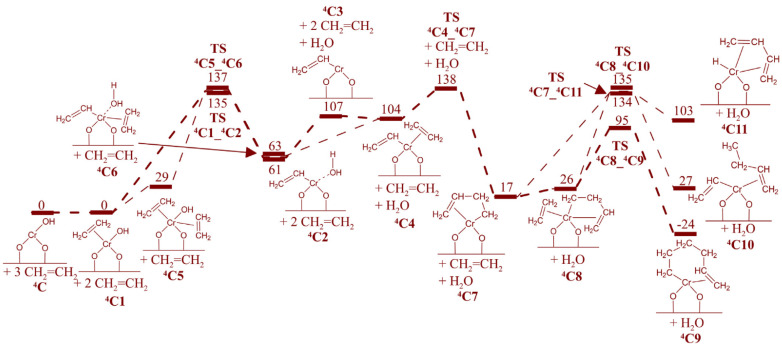
Gibbs energy profile (kJ∙mol^−1^) at T = 373 K for the mechanism of ethylene polymerization over Cr(III)–OH species (quartet reaction pathway). The thicker line represents the preferred route. Reprinted with permission from [76]. Copyright (2017) Elsevier B. V.

Hydroxylated monomeric Cr(III) species **^4^C** can be formed by the hydration of **^4^B**. The ethylene polymerization energy profile, based on **^4^C**, is presented in Figure 29. This newly proposed mechanism involving the propagating of Cr(III) vinyl sites appears to be reasonable for explaining the polymerization activity of the Phillips catalyst despite the value of the Gibbs energy barrier (135 kJ∙mol^−1^) for the initiation stage, which is only marginally higher than the value estimated experimentally (120 kJ∙mol^−1^) [72]. Finally, the modeling of ethylene polymerization on Cr(V) species (Scheme 9, pathway **XIII**) was also conducted, but the value of the activation barrier was found to be higher in comparison with the **^4^C**-based route.

**Figure 30:**
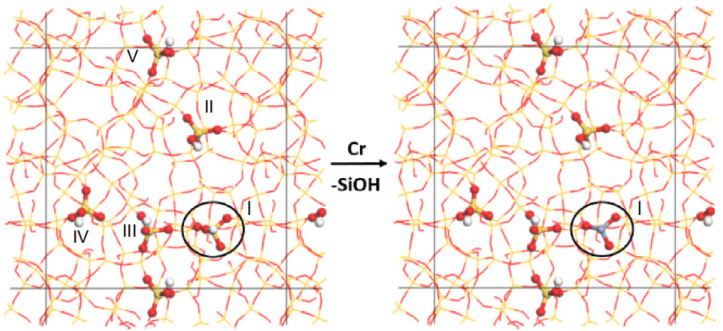
Top view of unit cell showing the five initial silanol groups (**I**–**V**). Construction of the Cr(III)/SiO_2_ model corresponding to model of Comas-Vives [26] by substitution of one of the five SiOH groups of the silica model with Cr. Reprinted with permission from [79]. Copyright (2017) Elsevier B. V.

Cr(III) oxide species three-fold bonded to the surface were studied in more detail by Copéret and colleagues [79] with the use of realistic amorphous periodic models that account for structural complexity, strain, and active site heterogeneity. They demonstrated that feasibility of the activation pathways **IX** (C–H activation) and **X** (insertion into Cr–O bond, see Scheme 9) depends critically on the Cr(III) environment. For the creation of the model that includes Cr sites, the visual method was used; namely, the silica model of Comas-Vives [26] with surface silanol density of 1.1 OH∙nm^−2^ was modified via substituting SiOH groups by Cr, which resulted in types **I**–**V** of (≡SiO)_3_Cr centers (Figure 30). 

**Figure 31:**
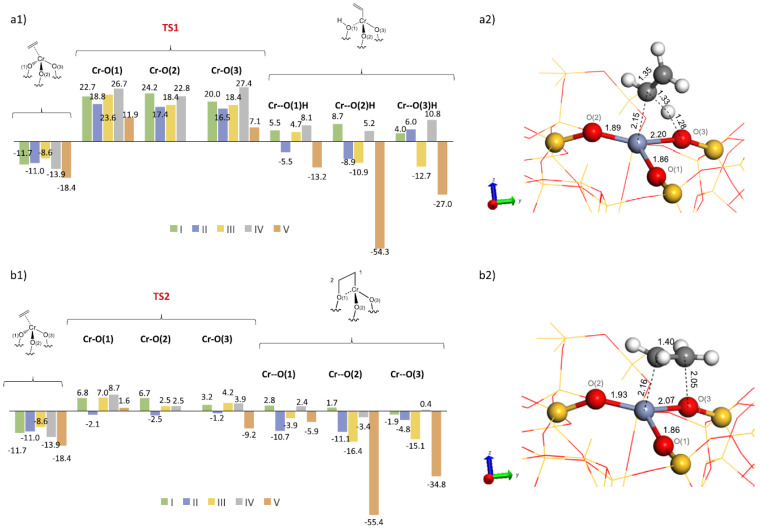
(**a1**) Energy profiles for C–H splitting of ethylene (kcal∙mol^−1^) as obtained with models **I**–**V** (1.1 OH∙nm^−2^). The energies are normalized with respect to the free system (bare model + ethylene). (**a2**) Zoom-in of the Cr(III) active site of model **V**. Transition state for C–H activation of ethylene on the Cr–O(3) bond. O–H–C_vinyl_ angle = 158.6°. Bond lengths (Å) are indicated. (**b1**) Energy profiles for insertion of ethylene into the Cr–O bond (kcal∙mol^−1^) as obtained with models **I**–**V** (1.1 OH∙nm^−2^). The energies are normalized with respect to the free system (bare model + ethylene). (**b2**) Zoom-in of the Cr(III) active site of model **V**. Transition state of the ethylene insertion into the Cr–O(3) bond. Reprinted with permission from [79]. Copyright (2017) Elsevier B. V.

The models **I**–**V** were first used to probe the reactivity of various Cr sites toward C–H bond activation of ethylene, and the results are shown in Figure 31a1. Coordination of ethylene to Cr(III) appears to be favorable in all cases. The energies associated with the products after the C–H bond activation of ethylene show a large degree of heterogeneity. They range from −54.3 to +10.8 kcal∙mol^−1^. An alternative mechanism via insertion of the C_2_H_4_ molecule (Figure 31b1) seems to be preferable; the presence of highly strained Cr(III) sites resulting from the thermal treatment of the support significantly facilitates both the C–H bond activation of ethylene and the insertion of ethylene into the Cr–O bond, leading to highly active sites and the formation of long-chain polymers. 

**Scheme 10:**
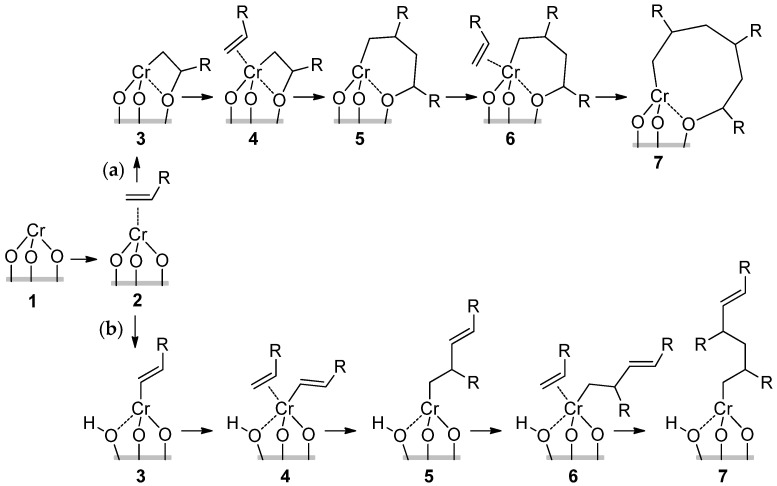
Evaluated alkene insertion (**a**) and alkene C–H activation (**b**) mechanisms in propene (R = CH_3_) and ethene (R = H) polymerization.

**Figure 32:**
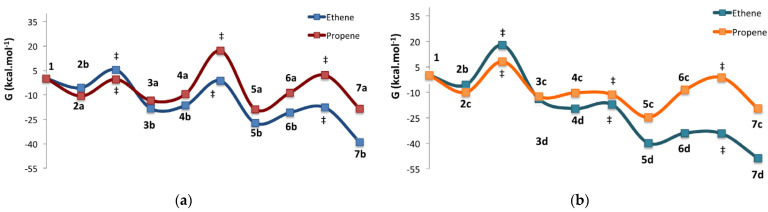
Gibbs energy profile (in kcal∙mol^−1^) for the (**a**) olefin insertion pathway (Scheme 10a) and (**b**) C–H activation pathway (Scheme 10b) for ethylene and propylene polymerization considering three alkene molecules. The symbols ‡ indicate the transition states. Reprinted with permission from [80]. Copyright (2017) Elsevier B. V.

As can be seen in Figure 31, the site **V** is the most active, and in [80], comparative simulations of ethylene and propylene polymerizations on this site were conducted in the frameworks of ethylene insertion and C–H bond activation mechanisms (Scheme 10a and b, respectively). As can be seen in Figure 32, alkene insertion into the formed Cr–C bond is particularly unfavorable for propene in comparison to ethene.

The possible role of chromacycle strain was estimated theoretically in a recent study of Núñez-Zarur and Comas-Vives [81] for the mechanisms presented in Scheme 10. Their results indicated higher activities of more strained Cr(III) sites and a preference for the mechanism of ethylene insertion into the Cr–O bond. The calculations were based on very simplified cluster models (up to seven atoms of silica with ≡SiH and even =SiH_2_ fragments), which have little in common with the real dehydrated Cr/SiO_2_ systems.

### 5.2. The Recent Comparative Results of the Spectroscopic Studies of the CrO_x_/SiO_2_ Activation

The data and discussion, presented in this section, also relate to the activation of Cr(II) species by ethylene. However, they address the common issues of the Cr(VI)/SiO_2_ activation based on experimental data and on the results of quantum chemical calculations of the molecular structures and spectra of the molecular Cr complexes, without any speculations regarding the reaction mechanisms. The starting point for actual investigations was the study of Torino’s group (E. Groppo and colleagues), who reported the first XANES/EXAFS in situ study of PCCs in ethylene polymerization [82]. 

**Figure 33:**
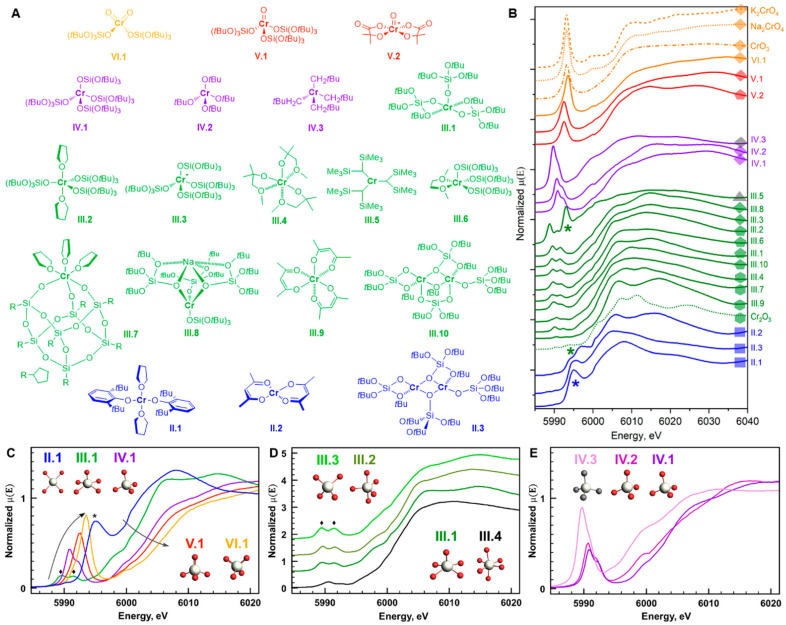
(**A**) Molecular Cr(II–VI) complexes investigated experimentally in [82]. (**B**) Experimental Cr K-edge XANES spectra of the series of crystalline reference complexes. Complex II.1 and III.5 display an intense shoulder (*) before the rising edge. Note that Cr2O3 shows a similar shoulder (*) in the pre-edge that arises from Cr–O–Cr long-range interactions and should therefore not be used as a reference for Cr(III) molecular complexes. (**C**) XANES spectra for selected molecular Cr complexes II.1–VI.1. Arrows indicate shifts of both the K-edge and pre-edge toward higher energy. Complex II.1 displays the intense shoulder (*) before the rising edge, and complex III.1 shows a characteristic splitting (◆) in the pre-edge. (**D**) Effects of ligand field changes on the pre-edge shape and intensity in III.1–III.4. The appearance of the weak pre-edge feature in the formally octahedral complexes III.4 (and III.7) is due to quadrupole transitions accompanied by small dipole contributions from distorted centrosymmetric ligand fields. All complexes show the characteristic pre-edge splitting (◆) independent of the ligand field. (**E**) Effects of the ligand σ-donor strength on the edge and pre-edge features of tetrahedrally coordinated Cr(IV) complexes IV.1–IV.3. Strongly σ-donating alkyl ligands show lower edge energies and higher pre-edge intensities. Reprinted with permission from [83]. Copyright (2021) American Chemical Society.

In 2021 [83], Copéret and colleagues reported the results of a study aimed at the explanation of the nature of PCCs at different stages (P1—Cr(VI)/SiO_2_, P2—CO-reduced precatalyst, P3—active catalyst) by analyzing the Cr K-edge XANES spectra for a series of tailored molecular Cr complexes varying in oxidation state, local coordination environment, and ligand nature and strength (Figure 33).

The quantitative analysis of the pre-edge region revealed the origin of the pre-edge shape and intensity distribution. In particular, the characteristic pre-edge splitting observed for Cr(III) and Cr(IV) molecular complexes was directly related to the electronic exchange interactions in the frontier orbitals (spin-up and spin-down transitions). The series of experimental references was extended by theoretical spectra for potential active site structures and used for training the extra trees machine learning algorithm. The most informative features of the spectra were selected as descriptors for the prediction of the Cr oxidation states, the mean interatomic distances in the first coordination sphere, and the type of the ligands. This set of descriptors was applied to uncover the site distribution in the Phillips catalyst at three different stages of the process, P1–P3 (Table 3).

According to the results of the study, the authors drew conclusions about the main oxidation states of Cr at different stages of their preparation and action: the freshly calcined catalyst consists of mainly Cr(VI) sites, the CO-exposed catalyst contains mainly Cr(II) with a minor fraction of Cr(III) sites, and the ethylene-exposed species contains mainly highly coordinated Cr(III) silicates along with unreduced Cr(VI) sites [83]. On the surface, these conclusions look reasonable, but the nature of P3 still appears indefinite, and the hypothesis about Cr(IV)’s nature of the active species seems to be still unrefuted.

Additional information about the nature of the active Cr(II) sites was obtained via comparative study of the molecular Cr complexes with and without ArNC ligands [84]; the results of the study allowed researchers to refine the process of CO coordination at Cr(II) centers.

To conclude this section, it should be noted that the use of *operando* XANES technique in combination with quantum chemical calculations and machine learning was the subject of a recent review [12].

### 5.3. Reaction with N_2_O

**Scheme 11:**
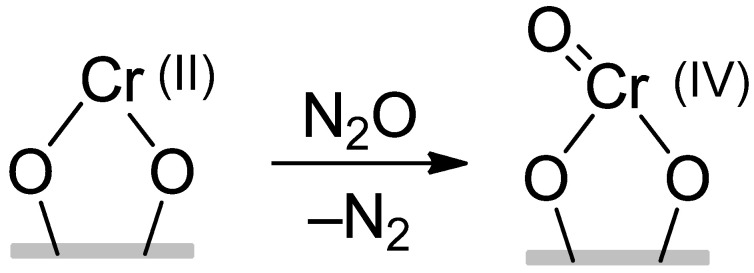
Hypothesis on the formation of Cr(IV) species during the reaction of Cr(II) sites with N_2_O [85].

Groppo et al. showed the high efficiency of the use of N_2_O for intermediate activation of Cr(II) sites [85]. They proposed that during the reaction with N_2_O, Cr(II) centers form a new type of the active Gr(IV) species (Scheme 11) that are able to coordinate and insert ethylene. Cr(II)/SiO_2_ was inert towards ethylene at room temperature, whereas after treatment by N_2_O at 100 °C, the catalyst showed high activity. Note that these results contradict the results of more recent work [47] but confirm the findings of McDaniel’s group when they studied the reduction of Cr(VI)/SiO_2_ by methane, which resulted in the formation of highly active Cr(IV) species [63].

### 5.4. Reaction with Silanes

Groppo and colleagues prepared CO-reduced PCCs and investigated the influence of SiH_4_ on the nature and activity of the catalytic sites [86]. They detected (but did not assign) new bands in FT-IR spectrum of SiH_4_-treated Cr(II)/SiO_2_ and showed that the SiH_4_-treated catalyst polymerizes ethylene seven times faster in comparison with the standard Cr(II) catalyst. In the first case, the product of ethylene homopolymerization had bimodal MWD with high content of –CH_3_ groups in low-MW polymer; the standard Cr(II) catalyst initiated the formation of HDPE. More thorough low-temperature FT-IR investigation confirmed in situ generation of α-olefins (mainly but-1-ene and hex-1-ene) by a SiH_4_-treated catalyst.

**Scheme 12:**
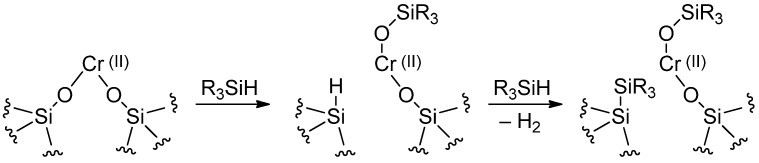
The two consecutive steps in the reaction between hydrosilanes and the Cr(II)/SiO_2_ catalyst [87].

In a continuation of the research [87], the interaction of Cr(II)/SiO_2_ with SiH_4_ and Et_3_SiH was studied by the analysis of the gaseous by-products, temperature- and pressure-resolved FT-IR spectroscopy, and deuterium exchange experiments. In the gas phase, only molecular H_2_ was detected. Taking into account the FT-IR spectral data, a two-stage mechanism was proposed (Scheme 12). The first stage involves the reaction of an R_3_SiH (R = H, Et) molecule with the (≡SiO)_2_Cr(II) site with a formation of the (≡SiO)Cr(II)–OSiR_3_ and ≡Si–H surface species. This latter species can successively react with a second R_3_SiH to give a ≡Si–SiR_3_ moiety and H_2_.

### 5.5. Reaction with Organioaluminum Compounds

Although R_3_Al and R_2_AlOR are used for the activation of Cr(VI)/SiO_2_ systems, their chemistry towards Cr(II)/SiO_2_ cannot be disregarded either. In 2018, Groppo and colleagues published a study of the effect of Et_2_AlOEt on Cr(II) sites, focusing their attention on the modification of the Cr(II) sites at a molecular level [88]. The Cr(II)/SiO_2_ species were prepared by impregnation of SiO_2_ (Aerosil^®^380) with a solution of chromic acid (with 0.5 and 1 wt% Cr loading) followed by degassing in dynamic vacuum with a temperature gradient to 650 °C, calcination at 650 °C, and reduction by CO at 350 °C. The treatment with Et_2_AlOEt was conducted in hexane media, with an [Al]/[Cr] ratio of 2:1. Blank SiO_2_ was also treated with Et_2_AlOEt for comparison.

Based on UV–vis spectral data, it was found that ~30% of the Cr(II) sites seemed involved in the reaction. At the same time, no specific features associable with the modified Cr sites were observed either in the UV–vis or in the FT-IR spectra. Estimation of the ethylene polymerization rates showed that modified sites were approximately 60 times more active in comparison with unmodified Cr(II)/SiO_2_. CO and MeCN probes revealed at least three types of Cr(II) sites with different reactivity. However, the presence of Cr–alkyl sites, resulting from organoaluminum treatment, remains unproven.

### 5.6. The Search for an Alternative Catalytic Species

Spectral evidence in favor of the formation of Cr(III)–CH=CH_2_ species via heterolytic C–H activation of ethylene was presented in the study of Copéret and colleagues [89]. In 2014, they reported the results of the study of the reaction of Cr(OSi(OtBu)_3_)_3_(THF)_2_ (**1**) with calcined silica in benzene with a formation of surface-bonded adduct **2** and its subsequent heating at 400 °C in vacuo with elimination of ~6 eq. C4 (isobutylene, *^t^*BuOH) and 1 eq. THF, resulting in the formation of species **3** [77]. Analysis of the extended X-ray absorption fine structures (EXAFS) of **1**–**3** (Table 4) confirmed the structure of **1**, specified the structure of **2** as (≡SiO)Cr(OSi(OtBu)_3_)_2_(THF), and allowed the researchers to determine the structure of **3** as (≡SiO)_3_Cr (some of these species were found to be weakly coordinated with a fourth oxygen atom). The presence of two types of Cr(III) sites in **3** was confirmed by the experiments on coordination of ^12^CO and ^13^CO. The polymerization activity of **3** was monitored at 70 °C and 0.325 bar ethylene pressure; PE with *M*_n_ = 33.6 kDa and *Ð*_M_ = 12.3 was formed. The IR spectrum of **3** contacted with ethylene contained the spectral bands at 3640 and 3605 cm^−1^, which are assigned to silanols interacting with adjacent Cr(III) sites to form Si–(μ-OH)–Cr(III) species, formed by heterolytic cleavage of a C–H bond of ethylene across a Cr–O bond.

**Scheme 13:**
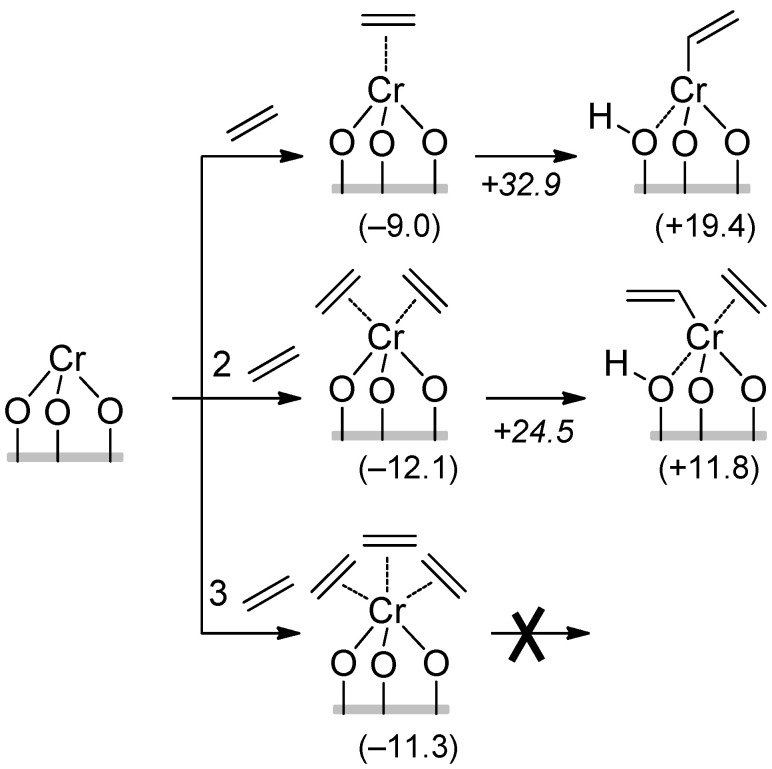
DFT study of the initiation mechanism of ethylene polymerization with **3**. Numbers in parentheses are Gibbs free energies of the corresponding intermediates and numbers in italics are Gibbs free energies of the corresponding transition states, normalized with respect to the system **3** + 4 ethylene (kcal∙mol^−1^) [77].

**Figure 34:**
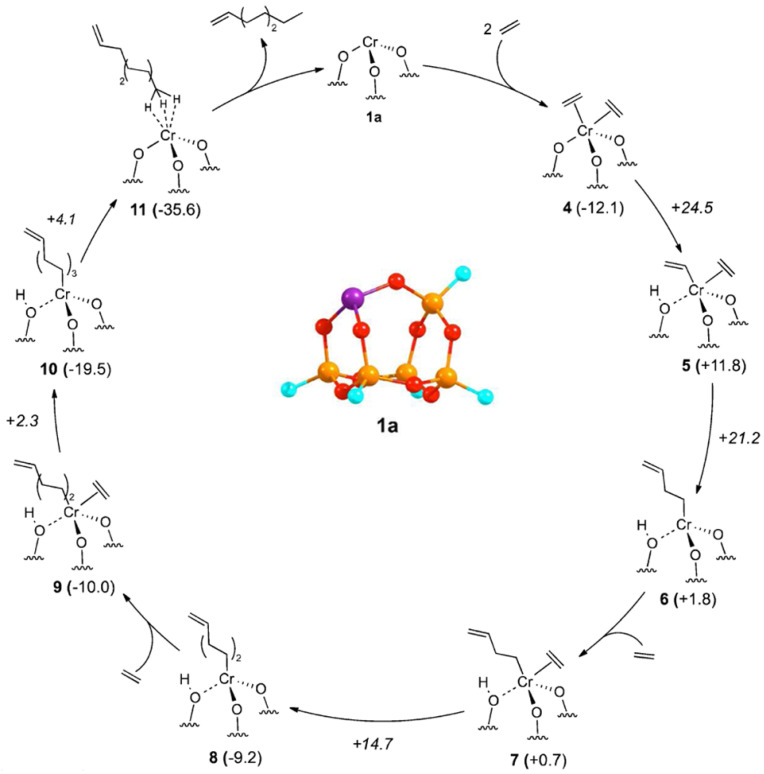
Ethylene polymerization mechanism by **1a**. The numbers in parentheses are the Gibbs free energies of the corresponding intermediate, and the numbers on the arrows are the Gibbs free energies of the transition states, normalized with respect to **1a** + ethylene. All energies are given in kcal∙mol^−1^. Reprinted with permission from [78]. Copyright (2015) American Chemical Society.

DFT modeling (B3LYP/BS1) of the coordination of one to three ethylene molecules and insertion of ethylene with a formation of Cr–CH=CH_2_ species was conducted using a simple cluster model containing five atoms of Si (see Figure 34) [77]. These calculations showed that coordination of two ethylene molecules with subsequent insertion (Scheme 13) appears to be feasible. Subsequent insertion of an ethylene molecule was found to be exergonic (−10 kcal∙mol^−1^) and associated with a low-energy transition state (<14 kcal∙mol^−1^). During further studies, ethylene polymerization on a Cr(III) cluster was analyzed theoretically in more detail [78] (Figure 34).

In the context of our review, it would be pertinent to mention here the comment of Theopold [90] about the studies of Copéret’s group [77]: the work “sets a high bar for those who would cling to more esoteric schemes for the formation of one of the most important industrial catalysts”. However, publication of [77] aroused much interest and discussion. In 2015, Peters et al. published an article containing significant corrections in the interpretation of the experimental data: they pointed out that the IR spectral bands at 3640 and 3605 cm^−1^ can be assigned to PE [91]. Another substantial concern related to the mechanism of chain release, in which termination via β-hydride transfer to ethylene with a formation of diene must also be taken into account. In their reply [92], Copéret and colleagues agreed with the comment about FT-IR identification of Si–(μ-OH)–Cr(III) species (adjusted in [78]); the question about the mechanism of chain release remained unclear.

Further theoretical studies of the formation of Cr(III)–CH=CH_2_ species using the same model cluster **1** (Figure 34) specified the influence of spectator ligands L in the complexes (≡SiO)_3_Cr(π-C_2_H_4_)L on the activation barriers of C–H scission [93]; it was shown that more donor ligands L facilitate this process; however, the ligand set was limited by CO, C_2_H_4_, N_2_, and C_2_F_4_ (which are shown in order of decreasing donor ability).

**Figure 35:**
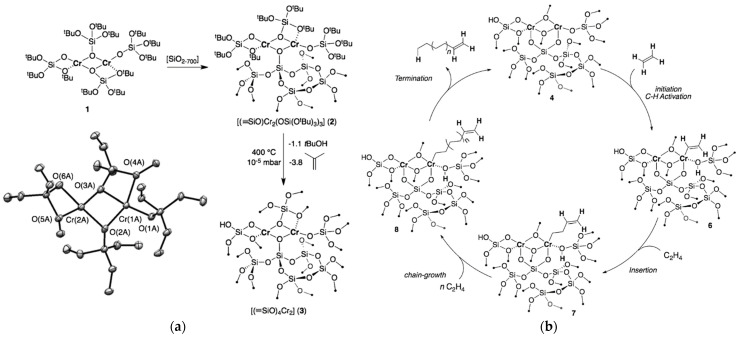
(**a**) Grafting of the complex **1** on SiO_2_ to form [(≡SiO)Cr_2_(OSi(O*^t^*Bu)_3_)_3_] and thermal decomposition under high vacuum to yield [(≡SiO)_4_Cr_2_] (**3**). The solid-state structure obtained by X-ray diffraction for an independent molecule of **1** is shown at 50% probability; (**b**) Proposed mechanism for ethylene polymerization by [(≡SiO)_6_Cr_2_]. Reprinted with permission from [94]. Copyright (2014) VCH Verlag GmbH & Co.

Dinuclear Cr sites, supported on silica, were studied by Copéret and colleagues in 2014 [94]. In these studies, they used binuclear Cr(III) complexes for the grafting into a SiO_2_ surface and showed that these species can provide ethylene polymerization (Figure 35a). Similar Cr(II) species were found to be inactive. Also, these studies showed the formation of a Si–(μ-OH)–Cr(III) species, consistent with an initiation mechanism involving the heterolytic activation of ethylene at Cr(III)–O bonds. However, in the corrigendum to this work [94], FT-IR spectral identification of these species was recognized as impossible.

To understand the nature of the catalytic sites in the real activated PCCs, a Cr[CH(SiMe_3_)_2_]_3_/SiO_2_ system was studied recently. In 2021, Torino’s group reported the results of the study of Cr[CH(SiMe_3_)_2_]_3_/SiO_2_ catalysts prepared on silica precalcined at 600 °C and differing in the chromium loading (0.1–0.5 wt.%) [95]. By applying DR UV–vis–NIR, X-band CW EPR, and IR spectroscopies, the two types of Cr(III) sites were discriminated: monografted ≡SiO–Cr[CH(SiMe_3_)_2_]_2_ species, responsible for ethylene oligomerization, and bis-grafted (≡SiO–)_2_CrCH(SiMe_3_)_2_ species, responsible for ethylene polymerization. These findings were notably corrected in very recent work of the Copéret group [96]. Using partially dehydroxylated silica thermally treated at 700 °C, which contains mostly isolated OH groups and thus yields well-defined monografted surface species, they detected monografted ≡SiO–Cr[CH(SiMe_3_)_2_]_2_ species in both high-spin (HS) and low-spin (LS) states, stabilized by additional interactions of Cr(III) centers with adjacent siloxane bridges; the LS monografted species were involved in ethylene polymerization.

To conclude this section, experiments on the preparation of a well-defined planar system modeling the PCCs using Cr physical vapor deposition on a metal-supported atomically flat silicate film [97] should be mentioned. These complex experiments have brought nothing new to the understanding of the mechanism of polymerization on PCCs.

## 6. Ethylene Polymerization and Copolymerization on PCC

### 6.1. Mechanisms of Chain Propagation

**Scheme 14:**
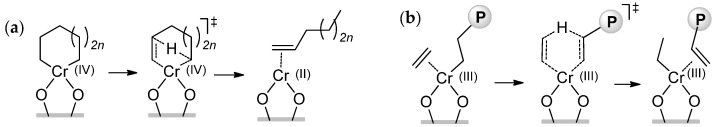
Chain termination for (**a**) metallacycle and (**b**) linear growth mechanisms of ethylene polymerization.

The unclear nature of the active center causes uncertainty about the polymerization mechanism. When comparing the metallacycle, Cossee–Arlman, and Green–Rooney mechanisms, the fundamental difference appears to be the chain termination event. The chain termination from a growing metallacycle proceeds through β-hydrogen transfer to the opposite end of the ring, whereas the Cossee–Arlman and Green–Rooney mechanisms involve a β-hydride transfer to a monomer that results in hydrogen scrambling between different oligomer/polymer units (Scheme 14). In 2010, McGuinness and colleagues reported the results of ethylene copolymerization with deuterated dec-1-ene and oligomerization of deuterated dec-1-ene, which revealed the hydrogen transfer between different polymer chains [98]. The study concluded that the metallacycle mechanism is not applicable to PE formation with PCC. To determine the preferred linear growth mechanism, copolymerization of C_2_H_4_ and C_2_D_4_ was studied; the value of the deuterium kinetic isotope effect k_H_/k_D_ was 1.04 ± 0.03, which indicates a preference for the Cossee–Arlman reaction pathway. 

**Figure 36:**
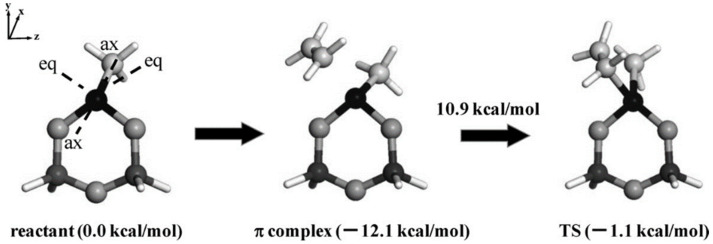
Ethylene insertion pathway for the simplest (≡SiO)_2_CrMe model. Black: Cr, dark gray: Si, gray: O, light gray: C, white: H. Reprinted with permission from [99]. Copyright (2011) Elsevier B. V.

The preference for the chain propagation for (≡SiO)_2_Cr^III^(alkyl) species in comparison with Cr(IV) metallacycles and (≡SiO)_2_Cr^IV^(alkyl)_2_ species was demonstrated by Tonosaki et al. through an example of the simplest models based on six-membered chromadisiloxane rings (Figure 36) [99].

### 6.2. Effect of Titania and Other Metal Oxides

Co-impregnation of Cr with the complexes of Ti and other metals has long been successfully used for the improvement of ethylene polymerization activity and branching control [3,8,100]. In 2016, Weckhuysen and colleagues [101] demonstrated that the higher Ti loading caused the appearance of more acidic hydroxyl groups and increased Lewis acidity of the Cr sites, thus shortening the induction time and increasing the initial polymerization rate. However, the nature of Ti centers on a silica surface was studied in depth very recently [102]; in the experimental (adsorption of polar molecules) and theoretical (QM/MM model of silica) work, two types of ≡Ti–OH sites were revealed, and more distorted sites with 15% content demonstrated higher Brønsted acidity.

In 2013 [103], Terano and colleagues reported the results of the comparative study of PCCs prepared by impregnation of Cr_3_(OH)_2_(CH_3_COO)_7_ and the complexes of other metals (see Table 5), taken in a molar ratio of 3:1, on the silica ES70X (*d* = 51 μm, SSA 320 m^2^∙g^−1^) in aqueous media. After drying and thermooxidative activation (O_2_, 400 °C, 2 h), the catalysts with 0.067 mol_Cr_∙g^−1^ and 0.022 mol_M_∙g^−1^ content were used in ethylene polymerization experiments (*n*-heptane, 70 °C, 5 bar ethylene). The influence of the second metal on the PCC’s activity was attributed to the change in the electron density of chromium centers (XPS data): less electronegative metals (Ti, Al, Zn, V, Zr) increased both activity and LCB formation, whereas more electronegative metals (Mn, Mo) reduced both PCC productivity and branching.

Studies of silica-supported mixed Cr/V oxide catalysts, prepared by impregnation/thermooxidative treatment using NH_4_VO_3_ and then basic chromium (III) acetate, were performed by Liu and colleagues [104,105]. After activation by ^i^Bu_3_Al, bimetallic catalysts demonstrated higher activities with a formation of bimodal polymers. More significantly, the bimodal ethylene/hex-1-ene copolymer showed “inverse” short-chain branch distribution in comparison with conventional Ziegler–Natta catalysts, with more comonomer incorporation into the high-MW PE chains formed on V catalytic centers. When using Et_2_AlCl for activation, UHMWPE was obtained but with very low activity [106]. In these works, only the results of polymerization experiments were discussed; the observed synergistic effect of the presence of Cr and V centers remained without mechanistic discussion. In [67], it was shown that the use of citric acid at the stage of impregnation of Cr and V salts resulted in more uniform distribution and high dispersion of the metal centers, which led to an increase in the activity and broadening of the MWD.

In similar work devoted to the study of mixed Cr/Mo oxide catalysts [107], a substantial decrease in catalytic activity was detected, which is consistent with the findings of Terano’s group; XPS data indicated an increase in the electron density of Cr species in the bimetallic catalyst.

An attempt to explain the difference between CrO_x_/SiO_2_ and VO_x_/SiO_2_ catalysts was made by Liu and colleagues in 2020 [108] with the use of the simplest possible metallasiloxane model, similar to the one shown in Figure 36. DFT calculations showed that the activation barriers for α-olefin insertion are lower for (≡SiO)_2_CrR species. It was found that the chain termination events may occur via both β-hydride elimination and β-hydride transfer for Cr(III) species, but for V(III) species, β-hydride transfer to a monomer is preferable. Th results are consistent with the experimental observations, viz., the formation of high-MW copolymers with higher α-olefin incorporation when using VO_x_/SiO_2_ catalyst. 

**Figure 37:**
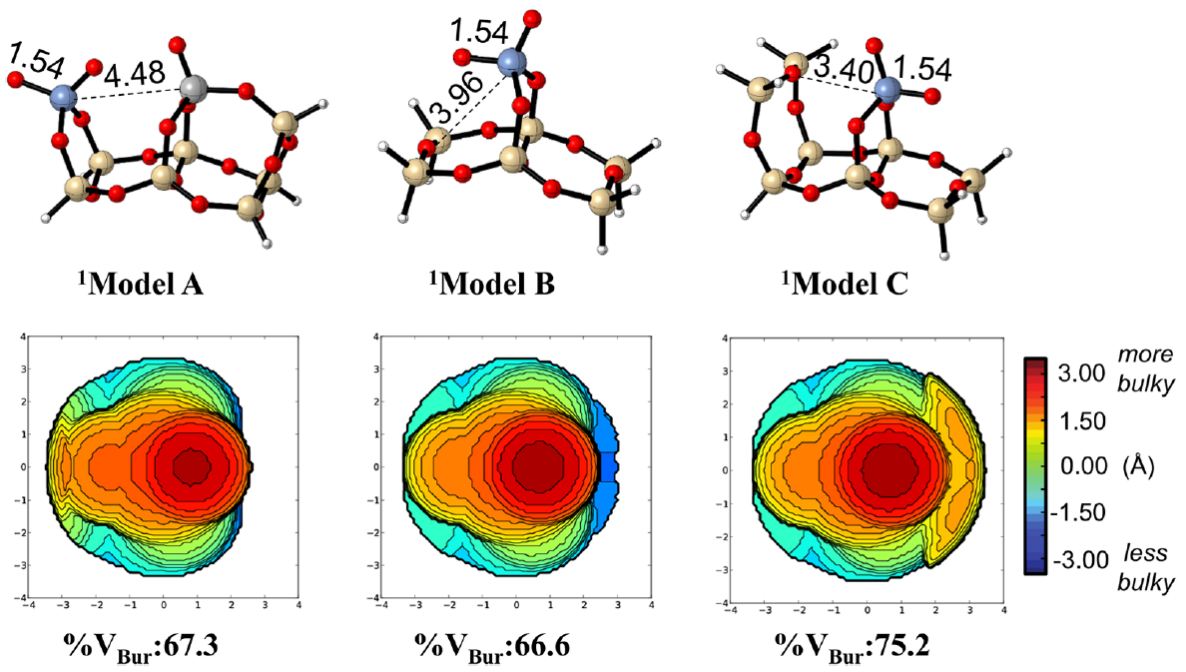
Geometric features of the optimized singlet model catalyst A, B, and C (oxygen atoms are in red; vanadium atoms are in gray; silicon atoms are in pale yellow; chromium atoms are in light blue). The active sites are oriented placing the metal center at the origin of the sphere. The top view of the steric maps is shown for three different active sites. Distances are given in Å. The steric hindrance maps of the intermediates were probed using SambVca 2.1. Reprinted with permission from [109]. Copyright (2021) Elsevier B. V.

A more detailed study of the Cr/V oxide catalysts [109] took into account the probability of the concerted Cr–V mechanism of ethylene polymerization. Three cluster models of the catalytic center were proposed (Figure 37); DFT optimization of the structures **A**–**C** and calculation of the buried volume %V_Bur_ values found no substantial difference in %V_Bur_ values for Cr–V species **A** and “opened” Cr species **B**. 

**Scheme 15:**
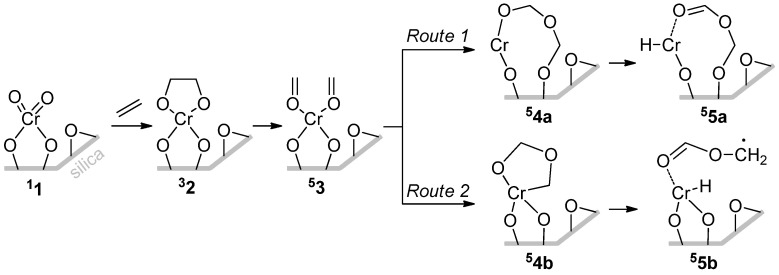
Proposed reduction and activation routes for PCCs with assistance of ethylene [109]. Route 1: reduction with a formation of six-membered cycle derivant; Route 2: reduction with a formation of five-membered cycle derivant.

DFT modeling of the activation of Cr(VI) species by ethylene was conducted in view of the proven HCOOMe formation [51,54] in the frameworks of the mechanisms presented in Scheme 15. In terms of Gibbs free energy, Route 1 has certain advantages compared with Route 2. The generation of free radicals also makes Route 2 less probable, and the breaking of the Cr–O bond seems to be essential for reduction. The relative Gibbs free energy values (Table 6) showed higher reactivity of species C at the stage of initiation.

**Figure 38:**
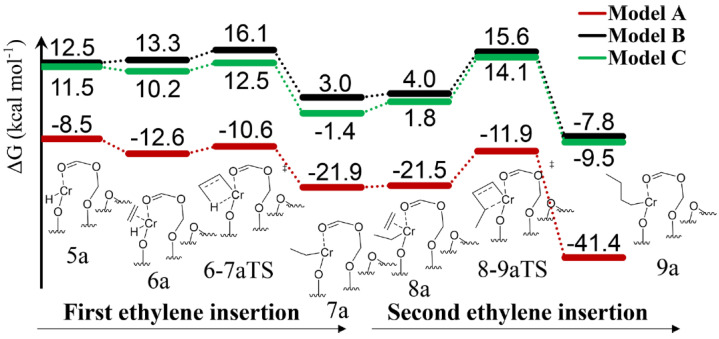
Further ethylene insertion of Route 1 over three models and distinction between Cr-H insertion and Cr-C insertion. The spin multiplicity for all complexes is quintet state. Reprinted with permission from [109]. Copyright (2021) Elsevier B. V.

The species **5a** seems to be an active intermediate state beneficial for further chain propagation. The mechanism of polymerization is presented as first (Cr–H) and second (Cr–C) ethylene insertions in Figure 38. The first ethylene insertion occurs with relatively low activation energies (2.0, 3.6, and 2.3 kcal∙mol^−1^ for **A**, **B**, and **C**, respectively). The energy barrier for the second ethylene insertion increases significantly (10.0, 12.6, and 15.5 kcal∙mol^−1^ for **A**, **B**, and **C**, respectively). Thus, ethylene insertion into the Cr–C bond is the rate-limiting step. Among the three models, Cr-V species **A** maintains the lowest activation energy for ethylene insertion.

**Scheme 16:**
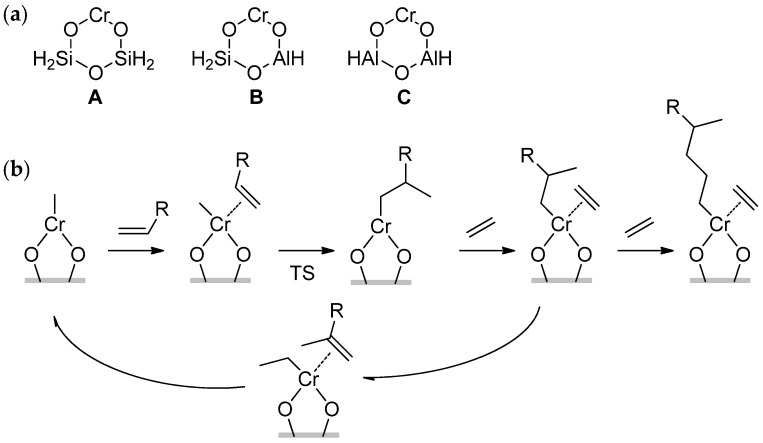
(**a**) Cluster models and (**b**) proposed chain propagation (R = *n*-butyl) and transfer pathway on cluster models through Cossee mechanism proposed in [110].

Industrially important Al-doped PCCs have been investigated extensively in recent years. In 2015, Liu and colleagues showed an increase in electron density of surface chromate species by Al modification [110], which was contrary to the results of Terano and colleagues [103]. However, the results of ethylene polymerization experiments showed that activity of the Al-doped PCCs was increased and the MW of the polymer was lower with the expansion of MWD to the low-MW side, which indicates the ease of β-hydrogen abstraction facilitated by a *decrease* in the electron density on the catalytic sites. An attempt of DFT modeling of the initiation and polymerization, based on simple CrO_3_Si_2_, CrO_3_SiAl, and CrO_3_Al_2_ models (**A**, **B**, and **C**, respectively, in Scheme 16a) seems to be too rough for the prediction of the properties of real catalysts. However, in part of the ethylene\hex-1-ene coordination and insertion in the frameworks of the Cossee–Arlman mechanism (Scheme 16b), DFT modeling allows us to draw some conclusions regarding the role of Al doping. As can be seen in Table 7, the chain propagation step is even more difficult after Al modification.

A small amount of titania, added to Cr/silica, can increase its activity several-fold (Table 8) [16].

### 6.3. Alkylaluminum-Activated Catalysts

One of the possible (and experimentally proven) roles of alkylaluminum activators is a formation of Cr sites that catalyze ethylene oligomerization with a formation of α-olefins that can further engage in polymerization with a formation of LLDPE. Among the works published in recent decades, the study of Weckhuysen and colleagues [111] with the use of commercial Cr/SiO_2_ samples, treated by Ti(O^i^Pr)_4_, deserves special mention. They showed that the combination of Ti and Et_3_Al results in the modification of the electronic environment of Cr oligomerization sites by the formation of Cr–O–Ti–O–Si linkages, increasing the selectivity towards but-1-ene formation at the expense of hex-1-ene. In the frameworks of the metallacyclic mechanism of ethylene oligomerization, it was proposed that an increase in Ti loading decreases the stability of the five-membered chromacyclopentane ring, favoring β-H transfer and the release of but-1-ene.

**Figure 39:**
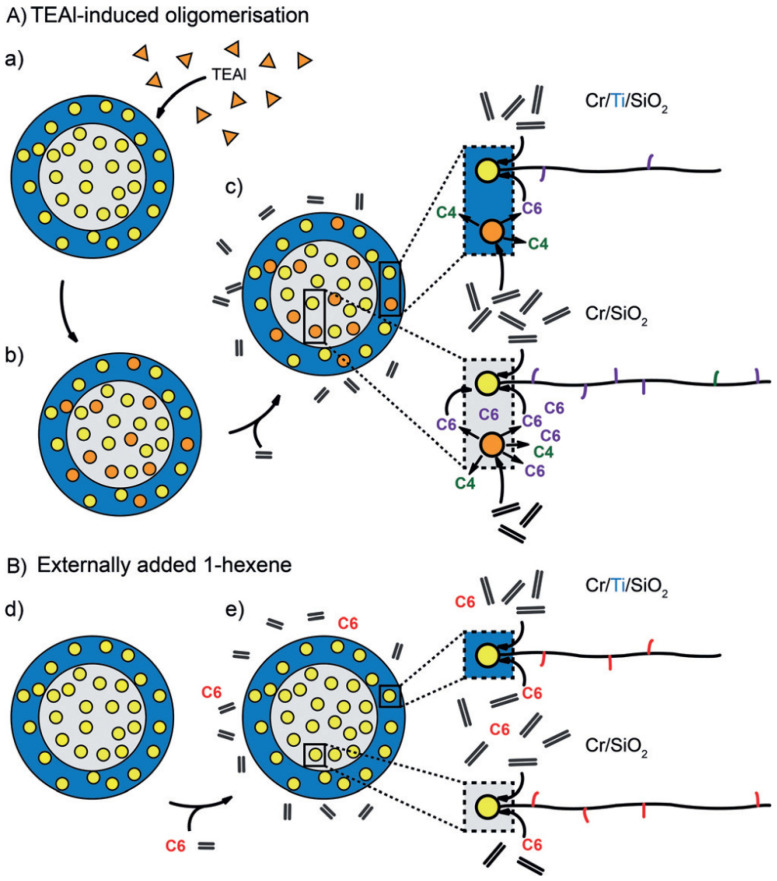
(**A**) Schematic representation of polymerization and oligomerization active sites of an Et_3_Al-modified shell–titanated Cr/Ti/SiO_2_ catalyst. (**a**) Et_3_Al (orange ▲) transforms some of the Cr polymerization sites (yellow ●) into (**b**) ethylene oligomerization sites (orange ●). (**c**) Oligomerization sites within the Ti-rich particle shell produce fewer oligomers. Therefore, nearby PE polymerization sites that make shorter chains in comparison to polymerization active sites within the Ti-scarce particle core incorporate a low amount of comonomer. Oligomerization sites within the Ti-scarce particle core are very efficient for the oligomerization of ethylene, producing 1-hexene predominantly. Hence, PE polymerization sites close by, which produce longer chains in comparison to polymerization active sites close to Ti, incorporate a higher amount of comonomer. This catalyst generates polyethylene with more comonomer in the long chains, that is, reverse comonomer incorporation. (**B**) If 1-hexene is externally added into the reactor using a pristine Cr/Ti/SiO_2_ catalyst (**d**), comonomer concentration is similar for all active sites, which leads to a typical Cr-based polyethylene with more comonomers in short chains (**e**). Reprinted with permission from [112]. Copyright (2015) Wiley-VCH Verlag GmbH & Co.

A remarkable example of manipulations with the nature of the catalytic species and polymer architectures was a further study of Et_3_Al-modified Cr/Ti/SiO_2_ catalyst by Weckhuysen and colleagues [112]. Since the Cr/Ti/SiO_2_ sites give low-MW polymer and have low comonomer reactivity [100], a research team from the Netherlands developed a new type of Phillips Cr/Ti/SiO_2_ catalyst, prepared from a commercially available Cr/SiO_2_ precatalyst by selective surface titanation on the catalyst particle’s outer shell and subsequent activation. As a result of treatment with Et_3_Al, a certain number of Cr *oligomerization* sites was formed, and this catalyst polymerized ethylene with a formation of a bimodal product containing low-MW linear PE (produced on Cr/Ti centers on the surface) and high-MW branched PE (formed on Cr polymerization sites from ethylene and ethylene oligomers in situ), as shown in Figure 39. The role of Et_3_Al in ethylene polymerization on Cr/Ti/SiO_2_ catalysts was studied in a separate work of this scientific group [101]. They proposed that it might be possible that Et_3_Al could break a Cr–O bond with the silica support so that a –OAlEt_2_ ligand is introduced instead of one rigid surface siloxy bond—in a similar way as the action of silanes. As a result, ethylene oligomerization Cr sites may be formed. 

### 6.4. Copolymerization and Comonomer Effect

The “comonomer effect” is a curious phenomenon in which the rate of ethylene polymerization is significantly enhanced by the addition of a small amount of α-olefin comonomer. The similar response is surprising because α-olefins incorporate into polyethylene chains much slower compared to ethylene. As applied to PCCs, the comonomer effect was studied and discussed relatively recently by McDaniel and colleagues [113]. In this work, they critically examined the theories that have been advanced to explain the comonomer effect on PCCs, which are (i) enhanced ethylene diffusion through a less crystalline PE due to the incorporation of the comonomer itself; (ii) higher catalyst fragmentation due to α-olefin incorporation; (iii) enhanced reduction of the chromium species in the oxidized catalyst and/or activation of dormant sites; and (iv) enhanced ethylene insertion due to the coordination of the comonomer to the chromium sites (“trigger mechanism” that can also be applied for the explanation of the formation of LCB PE). The latter concept seemed to be the most plausible; however, it needed direct experimental data indicating the presence of the α-olefin in the coordination sphere of the active Cr sites.

In 2016, Barzan et al. reported the results of the study of catalytic and spectral properties of a Cr(II)/SiO_2_ system in the presence of the nonreactive olefin cyclohexene [114]. They showed that the presence of cyclohexene enhances the rate of homopolymerization of ethylene, propylene, and hex-1-ene up to a factor of 5. But even more to the point, in situ FT-IR and DR UV–vis spectroscopic studies showed that cyclohexene does not react with the Cr(II) sites but strongly interacts with them. The experiments with additional CO coordination showed that all Cr(II) sites coordinate cyclohexene, and the reaction with more strong donor NO resulted in release of cyclohexene. To clarify the role of cyclohexene coordination, the homopolymerization of ethylene-*d*_4_ was followed by FT-IR spectroscopy. These experiments showed the presence of two families of Cr(II) sites: (i) a small fraction of sites readily active in ethylene-*d*_4_ polymerization, which do not retain cyclohexene during the reaction; and (ii) a larger amount of inactive sites which retain cyclohexene at least during the first minutes of reaction. The removal of cyclohexene from the Cr(II) sites active in d-ethylene polymerization excludes the occurrence of a “trigger mechanism” during the whole polymerization process. In other words, the origin of the comonomer effect has to be searched in a modification of the molecular and electronic structure of the Cr(II) sites by the comonomer: the more electron-donor comonomer induces a molecular and electronic rearrangement of the Cr(II) sites, which favors the *first* ethylene insertion during the initiation of the process. It is quite possible that the role of cyclohexene at this stage is somewhat similar to the role of additional Si–O–Si donor fragments, proposed by Peters and colleagues, In order to reduce the activation barrier for Cr–C homolytic cleavage after oxidative insertion of ethylene at the Cr(II) center [72].

### 6.5. Fluorine Effect

**Figure 40:**
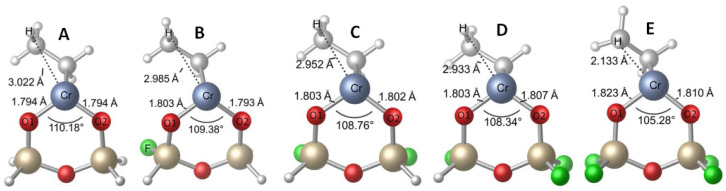
Geometric features of the optimized model catalysts (**A**–**E**). The bond lengths (Å) and the O1–Cr–O2 bond angles (°) are given. Reprinted with permission from [18]. Copyright (2017) Wiley-VCH Verlag GmbH & Co.

In 2017, Liu et al. reported the results of a comparative study of nonfluorinated and fluorinated PCCs [18]. Polymerization experiments showed that the addition of fluoride to the PCC can increase both the activity in ethylene homopolymerization and the molecular weight of the PE. In ethylene/hex-1-ene copolymerization, fluorinated PCC demonstrated higher activity and short-chain branch content in the high-MW copolymer fraction. These observations were confirmed by DFT calculations using a very simple model of the active Cr(III) species, (≡SiO)_2_Cr–Et (Figure 40).

**Scheme 17:**
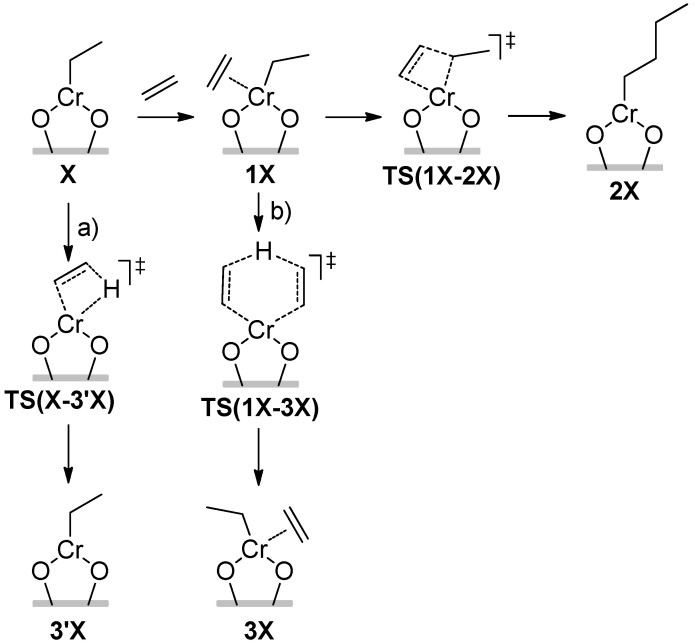
Chain propagation and chain termination. (a) β-hydride transfer to the Cr center and (b) β-hydride transfer to the incoming ethylene [18].

The difference in the geometry parameters depicted in Figure 40 shows that F-modification increases d(Cr–O1) and d(Cr–O2) and decreases the O1–Cr–O2 angle to result in a less strained chromasiloxane ring. The natural bond orbital (NBO) charges of Cr increase after F-modification (A: 1.129, B: 1.130, C: 1.131, D:1.138), but in the case of E, a strong β-agostic interaction lowered the NBO charge to 1.018. Further modeling of chain propagation and chain termination in the frameworks of the conventional Cosse–Arlman mechanism (Scheme 17) led to a conclusion that the presence of Si–F bonds in a chromasiloxane complex hinders the chain release via β-hydride transfer (Table 9). In ethylene/hex-1-ene copolymerization, the presence of F results in an increase in the difference between activation barriers of chain propagation and chain release after insertion of the hex-1-ene molecule. Note that activation barriers of β-hydride elimination (pathway (a) in Scheme 17) were not calculated, even though β-hydride transfer to metal is possible in theory [71].

In the same year, Liu et al. published the results of a study of fluorinated Cr/V catalysts [115]. Compared with a fluorine-free catalyst, the activities of fluorinated bimetallic catalysts slightly decreased with the increasing MW of the product, and the hydrogen response increased slightly.

A Cr(III)-alkyl Cossee–Arlman model was also used in a recent theoretical study of fluorinated PCCs [17]. Based on POSS structure without and with substitutions of Si–OH by Si–F, the reactivities of four-, six-, and eight-membered chromacycles were analyzed. It was shown that the presence of F increases the activity of eight-membered cyclic species; however, the relevance of both silica model and reaction mechanism for real PCCs seems doubtful.

**Scheme 18:**
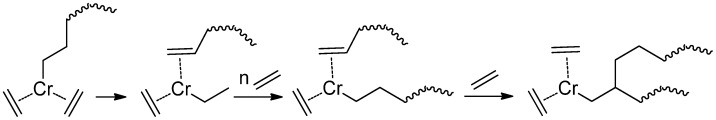
LCB formation by β-H elimination and later insertion of the macromer [16].

Another essential aspect is the use of more acidic supports in experimental observation of the higher LCB content in PE. McDaniel proposed that the increase in positive charge on the Cr center (in other words, an increase in electron deficiency) is thought to increase its attraction to α-olefins, thus increasing the chance of macromer retention and later insertion; the mechanism is illustrated in Scheme 18 [16]. It should be noted that a very similar scheme of the “intramolecular” LCB formation was proposed previously by McDaniel for zirconocenes [116] and confirmed by DFT modeling in our study of LCB PE formation, catalyzed by 5,10-dihydroindeno [1,2-*b*]indole-based constrained-geometry Ti(IV) complexes [117]. In addition, this mechanism proved to be inapplicable for [η^5^-C_5_Me_4_SiMe_2_N*^t^*Bu]TiCl_2_ experimentally [118] and theoretically [117].

## 7. Alternatives to Cr/SiO_2_ and Further Development of PCCs

### 7.1. Alternative Supports

Cr/Al_2_O_3_ catalysts have some drawbacks (e.g., lower fragility, porosity, and surface area, more rapid deactivation) in comparison with Cr/SiO_2_ systems [3,4]. However, the recent progress in the development of Al_2_O_3_-based supports [119,120] does not exclude the possibility of further studies and applications of alumina as applied to PCCs. In 2018, Groppo and colleagues conducted a separate study of the nature and activity of Cr sites on an alumina surface [121]. Amounts of 0.5 and 1 wt% Cr (in a form of CrO_3_) were impregnated on transition-Al_2_O_3_ Aeroxide Alu C (Evonik Industries, Essen, Germany) characterized by an SSA of 100 m^2^∙g^−1^, degassed in dynamic vacuum at increasing temperature up to 650 °C, oxidized at 650 °C in the presence of O_2_, and reduced by CO or H_2_ at 350 °C. UV–vis spectra showed that the reduction of Cr(VI)/Al_2_O_3_ leads to the formation of at least three types of reduced chromium sites, Cr(II)_4C_ (four-coordinated), Cr(II)_6C_, and Cr(III)_6C_. The reduction by H_2_ resulted in the formation of Cr(III)_6C_ (98%) and Cr(II)_4C_ (2%) sites, whereas reduction by CO lead to Cr(III)_6C_ (70%), Cr(II)_6C_ (22%), and Cr(II)_4C_ (8%) sites. The preferential formation of Cr(III) species during the reduction of Cr(VI)/Al_2_O_3_ distinguishes this system from Cr(VI)/SiO_2_. However, the surface species are Cr(II)_4C_, and only Cr(II)_4C_ demonstrated reactivity towards a CO probe. In addition, in contrast with Cr(II)/SiO_2_ systems, which are “naked” when using CO or H_2_ as reducing agents, a fraction of Cr(II)_4C_/Al_2_O_3_ species were found to be in proximity with surface –OH groups (H_2_) or carbonates (CO).

Comparative studies on ethylene polymerization showed that the CO-reduced Cr(II)/Al_2_O_3_ catalyst is almost 15 times more active than Cr(II)/SiO_2_; the productivity of H_2_-reduced Cr(II)/Al_2_O_3_ was 350 times lower in comparison with CO-reduced Cr(II)/Al_2_O_3_. However, the nature of the activated Cr(II) sites remained unclear in the study, and the participation of active Cr(III) species cannot be ruled out.

Evidently, the main drawbacks of alumina can be explained by the close proximity of the crystal cell parameters of Al(III) and Cr(III) oxides; the content of active Cr(II)_4C_ sites is low, and the main part of Cr simply integrates into the alumina structure as Cr(III) ions. However, the positive impact of the higher Lewis acidity of Al in comparison with Si cannot be ignored and might be used in further development of mixed inorganic phases as new-generation supports for PCCs.

### 7.2. Hybrid Catalysts

In 2011, Moreno et al. proposed the use of Cr(VI) forms of PCC, prepared from different inorganic phases (calcined silica, silica–alumina, aluminophosphate, and SBA-15) as supports for a (*^n^*BuCp)_2_ZrCl_2_/MAO system [122]. When silica–alumina was used as a support, the hybrid catalyst demonstrated similar activity in comparison with a metallocene catalyst. However, in all experiments, PEs with narrower MWD were obtained, with the main contribution of “metallocene” mode. These results are not surprising in light of the detrimental impact of organoaluminum compounds on the activity of CrO_x_/SiO_2_ systems. The use of a hybrid catalyst system containing PCC and Ziegler–Natta catalysts for the production of polymodal PE is protected by the Univation patent [123]. However, these hybrid catalysts were significantly inferior in activity to the Ziegler–Natta catalyst alone. Furthermore, as with PCC/metallocene systems, PEs with narrower MWD were formed.

The development of hybrid catalysts is a very attractive idea, but the compatibility of the desired components (R_3_Al and donors for Ziegler–Natta catalysts, MAO or R_3_Al/perfluoroaryl borates for single-site systems, no additives for PCCs) needs to be considered during the formulation process. Some of these additives are mutually incompatible, and it is hardly surprising that only Ziegler–Natta/metallocene hybrid catalysts have demonstrated real prospects to put into practice [124,125] with the use of “activating” supports [120].

**Scheme 19:**
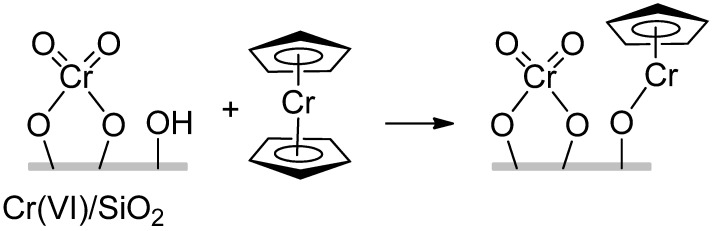
Preparation procedure of the SiO_2_-supported chromocene/CrOx dual-site catalysts.

In 2020, Liu and colleagues prepared a hybrid catalyst using the residual Si–OH groups on the surface of Cr(VI)/SiO_2_ to introduce (η^5^-C_5_H_5_)_2_Cr [126] (Scheme 19). The very idea of this type of catalyst is far from new, and silica is not the optimal support for chromocene-based catalysts [127]. The main problems of the similar-type hybrides is the need for R_3_Al activators and low thermal stability of supported chromocene; the optimal polymerization temperature was 60 °C. However, these systems seem prospective for the preparation of in-reactor blends of UHMWPE formed on the chromocene component, with HDPE produced on R_3_Al-activated CrO_x_/SiO_2_.

## 8. Conclusions

**Figure 41:**
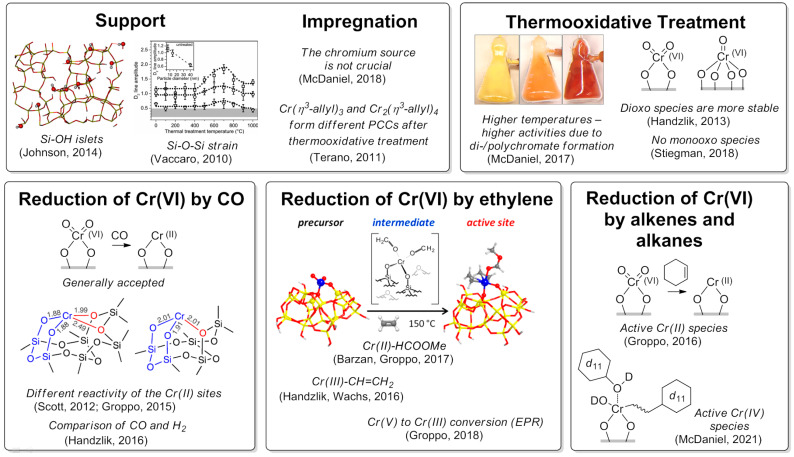
The most important aspects and contradictions of the stages of preparation and activation of Cr(VI)/SiO_2_ [3,25,28,33,36,39,40,42,48,49,50,54,55,58,62].

To sum up, the preparation and action of PCCs have been studied extensively for more than seven decades [3,4,5,6,8,9,10]. Since 2010, numerous experimental and theoretical studies have been performed; the scientific groups of C. Copéret, E. Groppo, and A. Zecchina (Torino group), J. Handzlik, B. Liu, M. McDaniel, S. Scott, M. Terano, F. Tielens, and B. Weckhuysen made the main contributions to recent investigations of PCCs. To date, a greater understanding of the mechanisms of Cr(VI)/SiO_2_ activation and action has been reached. The brightest and most interesting results of recent studies and actual problems and contradictions are presented in Figure 41.

However, the review of the progress and prospects of PCCs would not be complete without a mention of the studies of amorphous silica, the support for the preparation of PCCs. Experimental and theoretical works of the groups of G. Vaccaro [28], J. Johnson [25], A. Comas-Vives [26], and C. Li [29] can be considered as a basis for the further development of comprehensive and valid models of CrO_x_/SiO_2_ catalytic systems and the design of PCCs.

At first sight, the stage of the thermooxidative treatment of Cr-impregnated silica is quite clear: due to the high thermal stability of the silyl chromates, dioxo and monooxo species can be formed. However, the results of the studies of the Handzlik [39] and Stiegman [42] groups contradict each other (Figure 41). Note that Stiegman and colleagues showed that the introduction of Al into a silica surface may lead to an increase in the content of monooxo Cr(VI) sites [43]. The mobility of Cr(VI) was clearly demonstrated by McDaniel in a revealing experiment [36], but the study of the Terrano group [33] demonstrated the difference in catalytic behavior of the catalysts formed by mono- and binuclear Cr complexes that were subjected to thermooxidative treatment (Figure 41).

The reaction of Cr(VI)/SiO_2_ with CO does not raise questions; the formation of Cr(II) species with elimination of chemically inert CO_2_ is a generally accepted fact. However, the results of the study of this process revealed the formation of the Cr(II) sites with different environments and reactivities [48,49]. In our review, we decided not to separate the reduction of the Cr(VI)/SiO_2_ by H_2_, but the marked difference between CO and H_2_ [50] is also mentioned in Figure 41.

In early works, the activation of Cr(VI) sites by ethylene and CO are viewed as similar processes; however, the results of recent studies showed that these processes are different in their mechanisms of activation. In the frameworks of the common view on the reaction of Cr(VI)/SiO_2_ with ethylene, this process was initially considered as an even-electron transfer with a formation of Cr(II) species and CH_2_O. The lack of CH_2_O detection stimulated Torino’s group to study the reaction of Cr(VI)/SiO_2_ with ethylene [54], and HCOOMe was found to be the most probable oxygenated by-product, coordinated at the Cr(II) species. However, almost at the same time, Handzlik and Wachs detected the formation of Cr(III)–CH=CH_2_ using in situ FT-IR spectroscopy [40]. The presence of Cr(III) species in the products of the reaction of Cr(VI)/SiO_2_ with ethylene was detected using EPR spectroscopy by Groppo and colleagues [55], but they attributed it to the reduction of Cr (V); when using Cr(II)/SiO_2_, the ethylene polymerized without EPR evidence of the formation of Cr(III). Less than 0.5% of Cr(III) in an active catalyst, obtained via the reduction of Cr(VI)/SiO_2_ by cyclohexene [58], does not add clarity to the fundamental question about the oxidation state of the active Cr species obtained by reduction of Cr(VI)/SiO_2_ by olefins. In concluding the discussion of the Cr(VI)/SiO_2_ activation, the recent striking study of McDaniel and colleagues [62] should be mentioned. In their work, the reaction of Cr(VI)/SiO_2_ with saturated hydrocarbons was investigated, and the presence of alkyl in growing PE chains is an essential argument in favor of the formation of EPR-silent Cr(IV) active species (Figure 41); also note that Cr(II) and Cr(IV) active sites were detected by Groppo and colleagues in the products of the reduction of Cr(VI)/SiO_2_ by Et_3_Al [65].

**Scheme 20:**
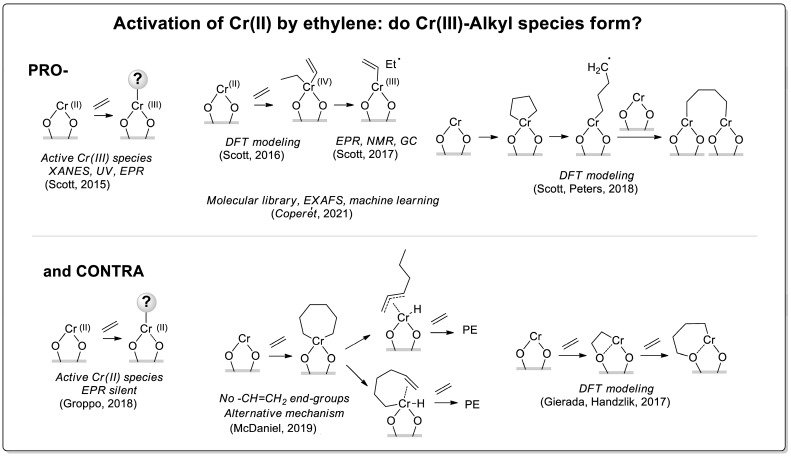
Experimental and theoretical results affirming and denying the formation of Cr(III)–alkyl species during reaction of Cr(II)/SiO_2_ with ethylene [47,55,66,72,74,75,76,83].

Perhaps the most striking and controversial experimental and theoretical results and their interpretations are presented in works devoted to the activation of Cr(II)/SiO_2_ species by ethylene (Scheme 20). The formation of Cr(III) sites during the stoichiometric “titration” of Cr(II)/SiO_2_ by ethylene at 80 °C was confirmed by *L*_2,3_-edge XANES, UV, and EPR spectral data by Scott and colleagues [47,74]. However, in the later work of Groppo and colleagues, the reaction of Cr(II)/SiO_2_ with ethylene was studied, including EPR monitoring, and an EPR-silent active polymerization catalyst was obtained [55].

Numerous attempts to solve the problem of “extra” hydrogen were often based on highly simplified models of Cr(II)/SiO_2_ species used for DFT simulations or were not supported by unambiguous experimental observations. To comply with the even-electron nature of the ethylene molecule, Scott and colleagues proposed the mechanism of the oxidative insertion of two ethylene molecules with a formation of (≡SiO)_2_Cr(CH=CH_2_)Et species and Cr(IV) sites, which may then undergo homolysis with a formation of Cr(III)-vinyl species [72]. The formation of (≡SiO)_2_Cr–CH=CH_2_ was detected by ^13^C CP-MAS NMR; *n*-butane was detected by GC [74]. But, again, this mechanistic concept, confirmed by spectral studies, could not withstand the experimental verification performed by McDaniel and colleagues in 2019 [66]: using CD_2_=CD_2_ at the initiation stage, they did not detect CD=CD_2_ end groups even in trace amounts, whereas saturated deuterated end groups were detected by ^2^H NMR. From the results of this study, a new initiation mechanism was proposed (Scheme 20). This mechanism involves the participation of the Cr(IV) active species.

In addition to the above-mentioned mechanistic concepts, a number of theoretical studies, both affirming and denying the formation of Cr(III)–alkyl species, have been conducted. One of them was the hypothesis of Scott and Peters about the formation of the binuclear alkylidene Cr(III) species [75]. In a more comprehensive theoretical study by Gierada and Handzlik [76], different Cr(II)- and Cr(III)-based mechanisms were analyzed. For Cr(II) species, an oxachromacyclic mechanism was proposed.

The use of artificial intelligence and machine learning is on the agenda; this fashion has even moved to the studies of PCCs. The recent attempt of deciphering PCCs by the formation of a library of Cr K-edge XANES spectra for a series of tailored molecular Cr complexes, quantitative analysis of the pre-edge region, calculations of the theoretical spectra, and use of these data for training the extra trees machine learning algorithm was performed by Copéret and colleagues In 2021 [83]. The conclusion was as follows: “The freshly calcined catalyst consists of mainly Cr(VI) sites. The CO-exposed catalyst contains mainly Cr(II) silicates with a minor fraction of Cr(III) sites. The Phillips catalyst exposed to ethylene contains mainly highly coordinated Cr(III) silicates along with unreduced Cr(VI) sites”.

An indirect argument supporting the Cr(III) nature of the catalytic centers in the reduced form of the PCCs is the high activity of the well-defined Cr–alkyl species obtained by the reaction of Cr[CH(SiMe_3_)_2_]_3_ with silica and the nearly identical characteristics of PEs obtained with the use of these catalysts and PEs produced with the use of PCCs [95,96]. However, the structure of the well-defined initiators ≡SiO–Cr[CH(SiMe_3_)_2_]_2_ and (≡SiO–)_2_CrCH(SiMe_3_)_2_ has little in common with the real nature of the active sites of PCCs—apparently, during polymerization, a well-defined initiator transforms to active species, which are still one of the “blackboxes” of coordination polymerization of ethylene and α-olefins.

Is it worth trying to “open” this box? Obviously yes, and further studies of PCCs may be performed with the use of new chemical probes, and the first shallow attempt was the use of ArNC ligands in a very recent study of Copéret’s group [83]. However, to avoid speculation and postmodern trends, we believe that the further investigation of PCCs should be guided by McDaniel’s principle: “to understand the catalyst, you have to make polymer”.

We think that, based on this principle, the following questions should be answered in further studies:

- Might strained SiOSi fragments be involved to the formation of immobilized chromium catalysts when using aprotic solvents and an appropriate chromium source?
- Is it possible to introduce the activating elements (Al, Ti, F) via the impregnation of the Cr complexes that include these elements in their composition?
- Is the use of polynuclear Cr complexes prospective for the preparation of next-generation PCCs?
- Since ethylene-reduced PCCs contain methyl formate, is it possible to fine-tune the catalyst by introducing other donors?

It is our belief that the further experimental and theoretical studies with the use of state-of-the-art approaches and methods will breathe new life into the development of PCCs.

## Figures and Tables

**Table 1:** Influence of Cr/silica calcination temperature on catalyst and polymer character [36].

Calcination Temperature, °C	400	500	600	700	800	900
[OH]∙nm^−2^ on virgin silica	2.42	1.80	1.41	1.13	0.92	0.76
Maximum [Cr(VI)]∙nm^−2^	2.3	1.5	1.2	0.9	0.6	0.5
% of Cr as dichromate at [Cr(VI)]∙nm^−2^ = 0.38	0	5	18	35	50	58
Activity, kg∙g^−1^∙h^−1^	0.4	1.1	2.0	3.3	4.0	5.7
MI, ^1^ g∙(10 min)^−1^	0.012	0.017	0.026	0.052	0.24	0.46
HLMI, ^2^ g∙(10 min)^−1^	0.94	1.05	1.65	3.31	13.99	26.10
*M*_w_, kDa	454	460	406	322	229	172
*Đ*_M_	37.1	28.4	14.9	12.3	7.6	6.9
LCB content, LCB∙10^−6^ C	1.4	1.5	1.8	2.7	7.7	9.8

^1^ Melt index, determined according to ASTM D1238 with a 2.16 kg load. ^2^ High-load melt index, determined according to ASTM D1238 with a 21.6 kg load.

**Table 2:** Catalyst properties and organic products obtained from the reduction of Cr(VI)/silica–titania catalysts [62].

Reductant	Color	Activity, ^1^ kg_PE_∙g^−1^∙h^−1^	Organic Products and Their Content, mol%
No	Orange	2.58–2.66	–
Methane	Green	4.54	MeOH (97); HCOOH (3)
Ethane	Blue	2.00	EtOH (98); AcOH (2)
*n*-pentane	Blue	3.19	Pentan-2&3-ols (46); pentan-2-one (25); pentan-1-ol (16); pentan-3-one (10); 2-penten-1-ol (2); pent-2-enal (1)
*n*-hexane	Blue	2.60	Hexan-2-ol (25); hexan-2-one (23); hexan-3-ol (20); hexan-3-one (17); hexan-1-ol (15)
Isobutane	Blue	n.d. ^2^	*t*-butanol (52); isobutanol (38); isobutanal (7)
i-pentane	Blue- green	n.d.	2-methylbutan-2-ol (30); 3-methylbutan-1-ol (20); 3-methylbutan-2-one (18); 2-methylbutan-1-ol (16); 3-methylbutan-2-ol (13); 3-methylbut-3-en-2-one (3)
Cyclopentane	Blue	n.d.	Cyclopentanol (75); cyclopentanone (17); cyclopentene oxide (6); cyclopent-2-en-1-one (2)
Cyclohexane	Blue	0.94	Cyclohexanol (65); cyclohexanone (27); Cyclohex-2-en-1-one (3); cyclohexene oxide (3)
Decalin	Blue	2.09	n.d.
Toluene	Dark blue	2.61	Benzaldehyde (42); benzophone-type dimers (29); Benzyl alcohol (17); 2&4-methylphenols (10)

^1^ Ethylene homopolymerization at 105 °C, gas phase, without additional solvent. ^2^ No data.

**Table 3:** Average Cr oxidation states from several independent approaches: LCF analysis of XANES spectra (pre-edge XANES region [5985–5997 eV] and the full XANES spectrum [5980–6100 eV] excluding pre-edge), pre-edge descriptors (area, centroid energy, and μ(E) max), and the prediction by the extra trees algorithm trained on 10,000 mixtures composed randomly from the reference library [83].

Method of Analysis	P1	P2	P3
LCF ^1^ of descriptors	5.5–6.0	2.0–2.5	3.2–4.0
Best pair for the LCF of descriptors ^2^	VI.1, V.3	II.6, III.6	III.7, VI.1
ML ^3^ prediction for descriptors	5.7–6.0	2.1–2.7	3.2–3.8
LCF of XANES (pre-edge)	5.6–6.0	2.0–2.5	3.5–4.1
Best pair for the LCF of the pre-edge	VI.1, IV.6	II.6, IV.2	III.7, VI.1
ML prediction for XANES (pre-edge)	5.6–6.0	2.1–2.7	3.4–4.0
LCF of XANES (postedge)	5.5–5.9	2.4–2.8	3.6–4.0
Best pair for the LCF of the postedge	Na_2_CrO_4_, V.1	II.3, IV.2	III.4, V.2/VI.2
ML prediction for XANES (postedge)	5.4–5.8	2.7–3.1	3.7–4.1

^1^ Linear combination fit. ^2^ See Figure 33. ^3^ Machine learning.

**Table 4:** EXAFS fit parameters for **1**, (≡SiO)Cr(OSi(OtBu)_3_)_2_(THF) **2**, and [(≡SiO)_3_Cr] **3** [77].

Sample	Neighboring Atom	Number of Neighboring Atoms	*r* (Å) ^1^	σ^2^(Å^2^) ^2^
**1**	O	3	1.866(5)	0.0049(9)
	O	2	2.018(5)	0.0049(9)
	C	4	2.97(1)	0.010(4)
	Si	3	3.553(8)	0.010(3)
**2**	O	3	1.95(3)	0.013(3)
	O	1	2.00(2)	0.003(2)
	C	2	3.02(2)	0.003(2)
	Si	3	3.2(1)	0.05(3)
**3**	O	3	1.973(9)	0.0027(8)
	O	0.4	2.50(6)	0.0027(8)
	Si	3	3.21(2)	0.009(2)

^1^ Distance between Cr and neighboring atom. ^2^ Debye–Waller factor.

**Table 5:** Effects of second metal on the ethylene polymerization activity and the branching frequency of obtained PE [103].

Second Metal Precursor	Activity, kg_PE_∙g^−1^∙h^−1^	Branch Frequency/10,000 C
–	0.79	0.43
Al(NO_3_)_3_	0.78	0.48
TiO(acac)_2_	0.85	0.51
V(acac)_3_	0.87	0.56
Mn(NO_3_)_2_	0.53	0.35
Fe(NO_3_)_3_	0.78	0.47
Co(NO_3_)_2_	0.72	0.46
Ni(NO_3_)_2_	0.80	0.35
Cu(NO_3_)_2_	0.81	0.47
Zn(NO_3_)_2_	0.86	0.50
ZrO(NO_3_)_2_	0.92	0.52
(NH_4_)_6_Mo_7_O_24_	0.65	0.45
(NH_4_)_6_H_2_W_12_O_40_	0.62	0.44

**Table 6:** Relative Gibbs free energy (kcal∙mol^−1^) for reduction over three models [109].

Structure ^1^	Model A	Model B	Model C
**^1^ 1**	0.0	0.0	0.0
**^1^ 2**	3.7	5.3	11.6
**^3^ 2**	−23.3	−23.2	−27.2
**^3^ 2–3 TS**	1.5	4.7	0.5
**^3^ 3**	−0.6	3.4	−1.1
**^5^ 3**	−23.8	−20.1	−19.5
**^5^ 3–4a TS**	−3.0	0.0	0.7
**^5^ 4a**	−21.7	−7.4	−5.3
**^5^ 4a–5a TS**	−0.7	14.1	13.8
**^5^ 5a**	−8.5	12.5	11.5
**^5^ 3–4b TS**	16.5	21.7	14.4
**^5^ 4b**	10.5	15.3	11.8
**^5^ 4b–5b TS**	34.2	37.1	39.1
**^5^ 5b**	23.3	27.3	32.6

Superscripts: ^1^—singlet state, ^3^—triplet state, ^5^—quintet state.

**Table 7:** Energy barriers (kcal∙mol^−1^) for chain propagation and transfer reactions on various models of Phillips catalysts [110].

Model	First Insertion (Ethylene or Comonomer)		Second Insertion (Ethylene)		β-H Shift to Ethylene		Δ(E_transfer_ − E_propagation_)	
	C_2_H_4_	1-C_6_H_12_	C_2_H_4_	1-C_6_H_12_	C_2_H_4_	1-C_6_H_12_	C_2_H_4_	1-C_6_H_12_
**A**	21.52	23.07	21.92	21.55	29.74	28.44	7.82	6.89
**B**	23.63	26.59	24.05	24.53	31.76	30.54	7.71	6.02
**C**	25.74	28.90	26.12	28.58	33.56	32.43	7.44	3.85

**Table 8:** The influence of titania, when applied to Cr/silica, on the catalyst activity in ethylene polymerization and resultant polymer properties [16] ^1^.

Titania [Ti]∙nm^−2^	Activity, kgPE∙g_Cat_^−1^∙h^−1^	*M*_w_, kDa	*Đ*_M_	LCB∙10^−6^ C
0.00	1.9	158	7.9	2.8
0.25	3.4	152	9.3	3.4
0.50	3.8	143	12.3	5.1
0.80	4.5	134	16.2	7.0
1.10	4.7	128	17.5	7.3
1.40	4.7	120	18.8	7.7
2.20	4.2	112	21.3	10.3

^1^ Calcination at 650 °C, polymerization at 105 °C (isobutane).

**Table 9:** Gibbs free energies of the first ethylene insertion into the Cr@C bond by the Cossee–Arlman mechanism and the corresponding chain transfer reactions over various models [18] ^1^.

Model	ΔG_298_ Relative to Separated Reactants [kcal∙mol^−1^]					Insertion Barrier ΔG^≠^_298_	β-H Transfer Barrier ΔG^≠^_298_	Δ ^2^
	1X	TS(1X-2X)	2X	TS(1X-3X)	3X			
**A**	−1.0	16.4	−10.4	23.7	−0.1	17.4	24.7	7.3
**B**	−1.8	16.1	−10.1	24.4	−0.1	17.9	26.2	8.3
**C**	−1.7	15.6	−10.2	24.8	−0.3	17.3	26.5	9.2
**D**	−2.5	15.0	−10.5	23.4	−1.1	17.5	25.9	8.4
**E**	−4.0	13.6	−11.4	22.3	−1.9	17.6	26.3	8.7

^1^ The Gibbs free energies are relative to the model catalyst and the corresponding number of free ethylene molecules. ^2^ The energy difference between the barrier for the β-H transfer and the barrier for the chain propagation.

## Data Availability

Not applicable.
